# Lung scRNA-seq reveals chronic inflammation and emphysemous phenotype in mice with osteogenesis imperfecta

**DOI:** 10.3389/fgene.2026.1713393

**Published:** 2026-02-26

**Authors:** Jennifer Zieba, Roya Bagheri, Alex Kot, Jorge H. Martin, Davis Wachtell, Sereen Wong, Maeve Mungovan, Caroline Wight, Deborah Krakow

**Affiliations:** 1 Department of Orthopaedic Surgery, David Geffen School of Medicine at University of California, Los Angeles, Los Angeles, CA, United States; 2 Department of Human Genetics, David Geffen School of Medicine at University of California, Los Angeles, Los Angeles, CA, United States; 3 Department of Psychology, University of California, Los Angeles, Los Angeles, CA, United States; 4 Department of Obstetrics and Gynecology, David Geffen School of Medicine at University of California, Los Angeles, Los Angeles, CA, United States; 5 Department of Pediatrics, David Geffen School of Medicine at University of California, Los Angeles, Los Angeles, CA, United States

**Keywords:** airway inflammation, cell-cell communication, emphysema, extracellular matrix, interstitial lung disease, osteogenesis imperfecta, single-cell RNA-sequencing

## Abstract

Osteogenesis imperfecta (OI), or brittle bone disease, is a rare congenital disorder characterized by bone fragility and increased fracture incidence mainly due to mutations in type I collagen or genes associated with collagen synthesis. Genetic and allelic heterogeneity underlie the phenotypic spectrum of OI yet all forms commonly feature early mortality stemming from pulmonary complications, the molecular cause for which has not been resolved. Using single-cell RNA sequencing (scRNAseq), we identified novel molecular and cellular mechanisms underlying the lung abnormalities observed in our *Col1a1* ^*Aga2/+*^ (*Aga2*) mouse, which recapitulates a moderate form of OI. Pulmonary tissues in OI models have consistently displayed a histological emphysematous phenotype, however the origin of this and the effect on lung cell development and function remains unknown. Using scRNAseq data derived from young and adult *Aga2* lungs, we found significantly increased AT2 to AT1 cell transition (cells necessary for alveolar structure and gas exchange) in young *Aga2* mice but decreased AT2 cell differentiation in adults. Further, adult *Aga2* lungs show increased fibroblast activation and differentiation. Finally, our scRNAseq analysis revealed a chronic inflammation phenotype in the Aga2 lung with increased neutrophil and monocyte numbers, IL1B and TNF pathway activation, NOD-like receptor signaling activation, and expression of the NLRP3 inflammasome. Most importantly, we saw a significant decrease in the expression of Scgb1a1 in immune, epithelial, and fibroblast cells. Decreased expression of Scgb1a1 is associated with multiple lung diseases such as emphysema, chronic obstructive pulmonary disease (COPD), and asthma and has become an important therapeutic target for chronic lung inflammation. Clinical treatments specific to pulmonary complications in OI are non-existent and our results reveal that chronic inflammation could be a target to prevent the pulmonary insufficiency and early mortality observed in OI patients.

## Introduction

Osteogenesis imperfecta (OI), also known as brittle bone disease, is a genetically heterogenous disorder resulting from mutations in at least 23 different genes (Forlino et al., 2011; Rauch and Glorieux, 2004). More than 85% of OI cases result from dominantly inherited mutations in *COL1A1* and *COL1A2* which encode the α1(I) and α2(I) chains of type I procollagen (Rauch and Glorieux, 2004; Byers and Pyott, 2012). The products of the remaining 21 known OI-associated genes act on type I procollagen biosynthesis, post-translational processing, osteoblast function, and bone mineralization. As genetic and allelic heterogeneity underlie the phenotypic spectrum of OI, variable expressivity has been seen for clinical features including limb contractures, muscle hypotonia, scleral hue, dentinogenesis imperfecta, hearing loss, as well as long bone and spinal deformities. While clinical severity is often described as mild, moderate, severe, and perinatal lethal, pulmonary abnormalities are recognized across the phenotypic spectrum. Pulmonary complications are the leading cause of mortality in OI, and respiratory deaths also increase with aging (McAllion and Paterson, 1996). Only recently has it been recommended that all OI patients be initially assessed for respiratory function via spirometry as part of routine clinical care (Tam et al., 2018).

Pulmonary compromise in OI has been attributed to secondary effects of scoliosis and rib fractures, leading to smaller ribcage volumes. However, recent studies reveal that patients without scoliosis or severe bone abnormalities have abnormal pulmonary function, suggesting that functional lung abnormalities are intrinsic to the disease (Thiele et al., 2012; Falvo et al., 1973). OI patients with respiratory compromise develop sleep apnea, shortness of breath, fatigue, insomnia, and are more susceptible to respiratory infections (McAllion and Paterson, 1996; Kang et al., 2017; Folkestad et al., 2016; Forlino and Marini, 2016; Mar et al., 2014; Himakhun et al., 2012; Li et al., 2002). Respiratory causes of death include acute and chronic respiratory failure due to vulnerability to viral and bacterial infections of upper and lower airways, chronic infection and development of bronchiectasis (McAllion and Paterson, 1996; Storoni et al., 2021). OI phenotypic features including short stature, chest wall abnormalities, scoliosis, immobility, muscle weakness and rib fractures can also exert negative effects on pulmonary function (Falvo et al., 1973; LoMauro et al., 2012; Widmann et al., 1999). Older descriptions of the lung in the perinatal lethal form of OI (OI type II) showed histological abnormalities including decreased alveolar number and immature development as well as pulmonary hypoplasia suggesting a developmental abnormality (Himakhun et al., 2012; Thibeault et al., 1995; Shapiro et al., 1989; Rodriguez et al., 1982). In recent years, intrinsic lung abnormalities have been uncovered using OI animal models demonstrating an effect on function (Thiele et al., 2012; Baglole et al., 2018; Grafe et al., 2014; Bald et al., 2010; Dimori et al., 2020).

Recently, *Tam et al.* systematically assessed lung function in a large cohort of OI patients spanning phenotypic severity, genotypes, gender, and ages while considering variables such as true height, mobility, scoliosis, and bisphosphonate use (Tam et al., 2018). This study revealed an overall reduction in forced vital capacity (FVC), the amount of air a person can exhale after inhaling fully, and forced expiratory volume (FEV_1_), the amount of air an individual can exhale in one second after maximal inhalation, measures of pulmonary function. From numerous studies in large “normal” cohorts, reduced FEV_1_ results in a significant increase in mortality even after adjusting for extrinsic factors such as age, sex, race, cholesterol levels, blood pressure, smoking status, alcohol consumption and BMI (Neas and Schwartz, 1998; Cohen et al., 1987). Further, patients with chronic lung diseases showing decreased FVC and FEV_1_ have an associated inability to respond to stressful situations such as pulmonary infections and surgery (McAllister et al., 2013; Singanayagam et al., 2013). While intrinsic and extrinsic factors contribute to pulmonary function, data implies that mutations leading to OI result in changes in lung morphology and function. This renders patients more susceptible to early mortality due to respiratory complications ranging from lung insufficiency to the inability to recover from extrinsic damage.

Several OI mouse models demonstrate lung abnormalities. Abnormalities shown by *Baldridge et al.* and *Dimori et al.* in the *Crtap* ^*−/−*^ mouse, recapitulating a severe OI phenotype, exhibited increased acinar air spaces (Bald et al., 2010; Dimori et al., 2020). *Dimori et al.* additionally performed respiratory mechanical and plethysmography measurements demonstrating functional abnormalities in *Crtap* ^*−/−*^ and *oim/oim* mice (loss of incorporation of the COL1A2 peptide into the type I collagen heterotrimer) (Dimori et al., 2020). Partial pressure-volume curves (P-V) in *Crtap* ^*−/−*^ mice had a flattened curve pattern similar to fibrosis and consistent with restrictive lung disease. However, when normalized to body weight, P-V curves showed patterns consistent with emphysema at higher pressures (Dimori et al., 2020). In the *oim/oim* mice, a more severe emphysematous pattern was seen both partial and high pressure scenarios. Lastly, the *Col1a1* ^*Jrt/+*^ mouse, a model of severe OI, displayed alveolar space enlargement (Baglole et al., 2018). These data indicate that OI mice with varying genotypes display a unique phenotype of restrictive and emphysema-like patterns and the observed morphological differences translate to lung function abnormalities. Interestingly, the human OI pulmonary study in *Thiele et al.* also showed evidence of both restrictive and obstructive patterns (Thiele et al., 2012), supporting the hypothesis that abnormalities in type I collagen produce morphological lung abnormalities and lead to altered function.

Collagen fibrils are the principal source of tensile strength in numerous tissues and properly synthesized and cross-linked collagen is integral to tissue integrity. Collagens are highly expressed in lung tissues and constitute a major portion of lung protein content (Suki et al., 1985). Fibrillar collagens including type I collagen have a major contribution to the overall architecture of the lung and are involved in alveolar formation and function (Faffe and Zin, 2009; Suki and Bates, 2008). Type I collagen is expressed by multiple lung cell types including fibroblasts, airway epithelial cells (AECs), and pericytes and plays major roles in early lung development/branching morphogenesis as well as postnatal alveolar function and maintenance (reviewed in Zhou et al. (2018)). Since most forms of OI stem from abnormalities in type I collagen, it is highly expressed in the lung and undergoes constant turnover throughout the life, it is not surprising to see pulmonary complications in OI patients. Yet the cellular mechanisms underlying them, and their functional consequences have not been well explored. Further, no specific treatment targets have been uncovered that could inform clinicians on how to approach pulmonary care.

In this study, we performed lung histology and single-cell RNAseq (ScRNAseq) analysis on postnatal day 5 (p5) and 28 (p28) wild-type (WT) and *Col1a1* ^*Aga2*^ (Aga2) lungs. The *Aga2* mouse model was generated by *Lisse et al.* via an ENU mutagenesis strategy that generated a frameshift mutation in the *Col1a1* gene, *Col1a1+/-* c.-16 T>A, exon 50, causing an extension of the COL1A1 protein that interferes with trimer formation (Lisse et al., 2008). The model is characterized by low bone mass and increased fracture incidence, with severity ranging from moderate to severe and has been used to study endoplasmic reticulum stress in OI (Lisse et al., 2008). Similar to previous lung studies conducted in other OI mouse models, at p28 we found increased alveolar spacing reflecting an emphysematous phenotype. RNA-seq analysis revealed that alveolar fibroblasts, which express the highest amount of type 1 collagen, showed increased expression of other fibrillar collagens as well as cellular differentiation markers. Aga2 epithelial cells exhibited changes in differentiation depending on the p5 and p28 timepoints resulting in alterations in epithelial cell numbers. Additionally, we saw significant increases in inflammatory cell numbers along with the differential expression of several pathways in alveolar macrophages important in lipid metabolism, the inflammatory response, and cell-cell communication. Aga2 fibroblasts and epithelial cells showed increased expression of several factors known to recruit inflammatory cells and contribute to pathogenesis in COPD and cystic fibrosis (Oz et al., 2022; Racanelli et al., 2018). The data support that chronic lung inflammation should be considered as a potential contributor to OI pulmonary dysfunction. Overall, to date, this study is an initial foray into the OI lung on a single cell level and introduces several mechanisms that could uncover why OI patients exhibit intrinsic lung insufficiency as well as exaggerated intolerance to environmental damage.

## Results

### Aga2 lungs show increased alveolar spacing at p28

**FIGURE 1:**
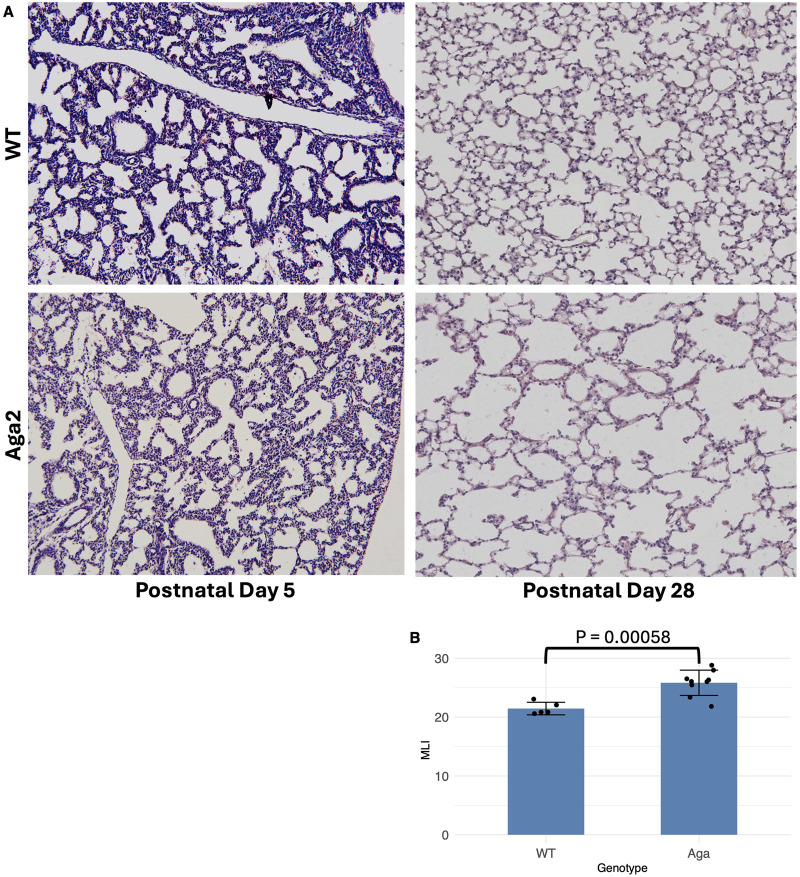
Aga2 mouse lungs show increased alveolar spacing at p28. **(A)** Paraffin sections of mouse lung stained with H&E. Top: Representative image of WT p5 and p28 lung. Bottom: Representative image of Aga2 p5 and p28 lung. Scale Bar = 100 μm. **(B)** MLI quantification showing increased alveolar spacing in Aga2 mouse lungs at p28. N = 5 WT, 9 Aga2.

To determine whether the Aga2 mouse showed a postnatal lung phenotype, we isolated lungs from p5 and p28 mice. Lungs were insufflated as described in the methods section. Sectioned lungs were stained with H&E. We consistently observed larger alveolar spacing in Aga2 mice at p28 when compared to WT lungs (Figure 1A). This was not observed in p5 lungs. To quantify this observation, we performed mean linear intercept (MLI) analyses and found a significant increase in MLI indicating increased alveolar spacing (Figure 1B). These results suggest an emphysemous lung phenotype at p28.

### ScRNAseq profiling of p5 and p28 Aga2 lungs

To determine the molecular changes behind the Aga2 lung phenotype, we performed single-cell RNA sequencing on both p5 and p28 Aga2 and WT lungs. We chose the p28 timepoint to analyze the consequences of the observed emphysemous phenotype and the p5 timepoint to determine whether there were molecular events earlier in postnatal lung development. Lung tissues were dissociated using a mixture of collagenase and dispase, single-cell 3′ library construction was performed using the Chromium platform, capturing approximately 10,000 cells per sample. Four mice were included in each group (WT and Aga2) and timepoint (p5 and p28) amounting to a total of 16 mice. WT and Aga2 samples were collected and similarly processed over several days. We did not find sex-specific gene expression changes in our analyses and thus combined both sexes in the analysis (Supplementary File S1, Supplementary Figures S14, S15). An average of 3.8 million reads were sequenced with an average of 41,000 reads per cell and over 20,000 genes mapped in an estimated total of 90,861 cells from all 16 samples.

**FIGURE 2:**
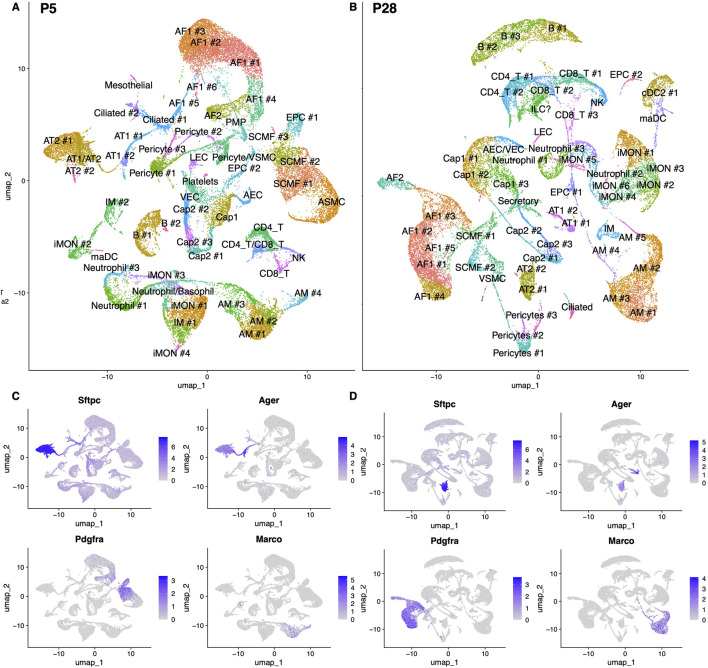
scRNA-seq analysis of p5 and p28 WT and Aga2 lungs. **(A,B)** Cell clusters from scRNA-Seq analysis visualized by uniform manifold approximation and projection (UMAP). **(A)** p5 timepoint, N = 4 for each genotype. **(B)** p28 timepoint, N = 4 for each genotype. **(C,D)** Feature plots showing the expression of lung cell markers: Sftpc (AT2 cells), Ager (AT1 cells), Pdgfra (fibroblasts), Marco (macrophages). **(C)** p5 timepoint. **(D)** p28 timepoint.

We used the Seurat package to perform principal component analysis and graph-based clustering using 20 dimensions and a 1.4 resolution after quality control filtering, subsetting, and normalization. We clustered the cells based on RNA expression and uniform manifold approximation and projection (UMAP) cluster analysis of the integrated single-cell data revealed 54 unique clusters (Figures 2A,B; Supplementary Figures S1, S2). We annotated the cell types using LungMap, a publicly available database of human and murine single cell RNA-seq and proteomic data, published in 2023 (Guo et al., 2023). Clusters were initially categorized into broad cell types using common lung markers that were highly expressed in a cluster when compared to all other clusters in the lung (Figures 2C,D). The clusters were then subtyped using top marker genes expressed in a cluster when compared to other clusters within the same category. Common lung markers and top sub cluster markers are listed in Supplementary Files S2,S3. Both broad and subtype clusters were used for downstream differential expression and communication analysis. The nomenclature and many of the markers used to delineate between celltypes and subtypes were derived from the LungMap database, which has become a standard in the field of lung single cell sequencing analysis (Guo et al., 2023). Additionally, if we found multiple subclusters within the same celltype, we numbered them sequentially according to cluster size with #1 being the largest subcluster.

We were able to delineate between multiple fibroblast populations: alveolar fibroblast 1 (AF1), alveolar fibroblast 2 (AF2), secondary crest myofibroblast cells (SCMF; myofibroblasts), and proliferative mesenchymal progenitors (PMP). Based on gene expression, we identified the AF1 cluster as a mixture of homeostatic fibroblasts, the AF2 cluster as activated matrix fibroblasts with the potential to differentiate into myofibroblasts, and the PMP cluster as a subset of fibroblasts enriched in cell cycle processes. The PMP cluster, a progenitor population, was only identified at p5 and not p28. Within alveolar fibroblast and myofibroblast groups, we found that each had several subclusters. In AF1 clusters there was a mixture of alveolar fibroblasts, lipofibroblasts, and adventitial fibroblasts at p5 and p28 with expression markers summarized in Supplementary File S3. In SCMF clusters, at p5, there were both developing and mature myofibroblasts. SCMF #1 expressed high levels of Fgf18 and designated as a mature SCMF population. SCMF clusters #2 and #3 contained early differentiated myofibroblasts derived from smooth muscle cells or alveolar fibroblasts, respectively. At p28, SCMF #2 expressed all the standard myofibroblast markers while SCMF #1 showed expression of several inflammatory myofibroblast markers including Coro1a and Ptprc.

Epithelial cells also showed multiple subclusters. AT2 cells secrete surfactant, are considered epithelial progenitor cells, and can differentiate into AT1 cells that are involved in gas exchange (Ohnishi et al., 2024). The AT2 #1 cell cluster was identified as activated AT2 cells with the high expression of Sftpc and Lcn2, an AT2 to AT1 transition marker. The AT2 #2 cell cluster expressed high levels of Sftpc as well as Cdk1 and Mki67 and was therefore designated as proliferative AT2 cells. The AT2 #1 cluster represented activated AT2 cells, likely to act as AT1 progenitors with higher expression of Il33 and Krt8, while the #2 cluster was designated as homeostatic AT2 cells with higher surfactant expression. At p5, clusters of epithelial cells expressed both AT1 and AT2 gene expression markers, Sftpc and Ager, as well as the transition marker Krt8 and thus was designated as subtype AT2/AT1 transition cells. We designated the AT1 #2 cell cluster as recently transitioned AT1 cells since they still express Krt8 but no longer express Sftpc. The AT1 #1 cell cluster represented mature AT1 cells with high expression of Ager and low expression of transition markers Krt8 and Trp53. By p28, the AT1 #1 cluster represented mature AT1 cells, whereas the #2 cluster represents recently differentiated cells with a higher expression of Scgb1a1.

Macrophage and monocyte clusters also separated into several subclusters. Clusters designated as alveolar macrophages (AMs), are tissue-resident macrophages with high expression of Cd206 and low expression of Cd86 among other known markers (Hou et al., 2021). The clusters named inflammatory monocytes (iMON) are monocyte derived macrophages with low expression of Cd206 and high expression of Cd86 that can differentiate into AMs. The interstitial macrophage (IM) cluster expressed high levels of Cx3cr1. Tissue resident AMs function in homeostasis and inflammation initiation whereas iMONs and IMs mainly secrete pro-inflammatory cytokines (Hou et al., 2021). Because alveolar macrophages show high plasticity and combined expression of classic macrophage markers, their heterogeneity has yet to be fully resolved (Hou et al., 2021; Mitsi et al., 2018).

### Type 1 collagen is highly expressed in activated matrix fibroblasts

**FIGURE 3:**
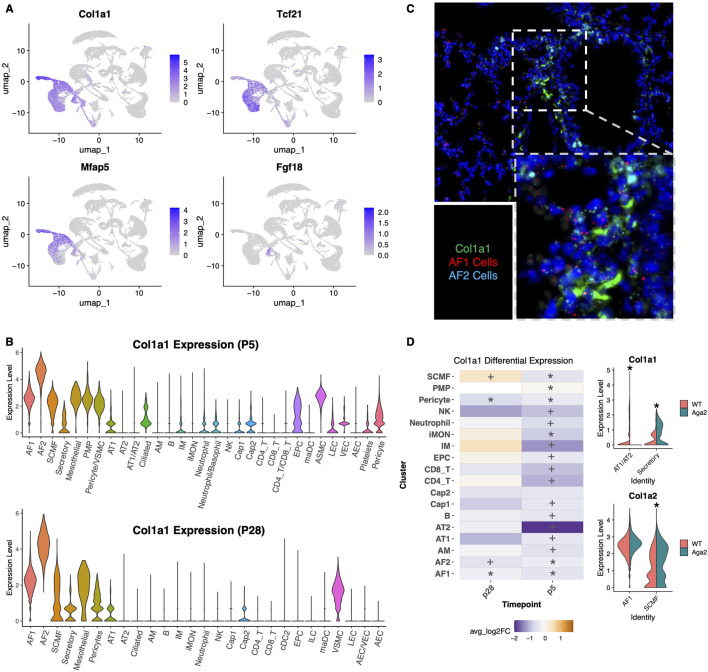
Type 1 collagen is highly expressed in alveolar fibroblasts and reduced in the Aga2 lung. **(A)** Feature plots showing the expression of fibroblast markers: Col1a1, Tcf21 (resting AF1 fibroblasts), Mfap5 (activated AF2 fibroblasts), Fgf18 (SCMFs) in p28 lungs. **(B)** Violin plots showing Col1a1 RNA expression in all identified lung cell types at p5 and p28 timepoints. **(C)** RNAscope for Col1a1 (green) costained with Tcf21 (red), Mfap5 (cyan), and DAPI (blue) indicating the presence of Col1a1 expression in both AF1 and AF2 cells mainly near lower airways in a WT p28 mouse lung. **(D)** Left: Heatmap showing differential expression of Col1a1 an Aga2 vs. WT mice in multiple lung cell types at p5 and p28. * = adjusted P value <0.05, + = non-adjust P value <0.05. Top right: Violin plot showing Col1a1 expression at p5 in AT1/AT2 and secretory/club cells indicating significantly decreased expression in Aga2 AT1/AT2 cells but increased in Aga2 secretory/club cells. * = adjusted P value <0.05. Bottom right: Violin plot showing significantly increased Col1a2 expression at p28 in Aga2 AF1 and SCMF cells. * = adjusted P value <0.05.

The expression of type I collagen is established in the developing and postnatal lung. Data derived from the LungMAP database, indicates that over time, type I collagen protein expression increases throughout the lung and is mainly expressed in lung myofibroblasts/mesenchymal cells and fibroblasts (Guo et al., 2023). To confirm this, we used our single cell data to determine the cell type with the highest expression of type I collagen. We found high expression of Col1a1 in matrix fibroblast clusters specifically in the activated fibroblast (AF2) cluster, a cell type that is known to support AT2 cell proliferation and differentiation during postnatal lung maturation and homeostasis (Figure 3) (Nabhan et al., 2018; McGowan and McCoy, 2014). Col1a1 was also expressed at lower levels in AF1 cells as well as myofibroblasts at both the p5 and p28 timepoint (Figures 3A,B). We performed RNAscope on paraffin sections of p28 WT mouse lungs to spatially determine the location of Col1a1 expression. The analysis showed that Col1a1 is expressed in AF1 and AF2 cells most highly near larger airways but also in distal alveolar areas, supporting the ScRNAseq data (Figure 3C).

At p5, Col1a1 RNA expression is significantly decreased in Aga2 alveolar fibroblasts and myofibroblasts, as well as PMPs, epithelial cells, and several immune cell types (Figure 3D). Col1a1 expression was also markedly decreased in Aga2 AT2/AT1 transition cells at p5 but significantly increased in secretory (club) cells. At p28, Col1a1 RNA expression was also decreased in AF1 and AF2 cells but slightly increased in SCMFs in the Aga2 lung. Interestingly, RNA expression of Col1a2 is significantly increased in alveolar fibroblasts at p28, also indicating a possible compensatory mechanism for reduced Col1a1 expression.

### Aga2 matrix fibroblasts show altered expression of differentiation markers

**FIGURE 4:**
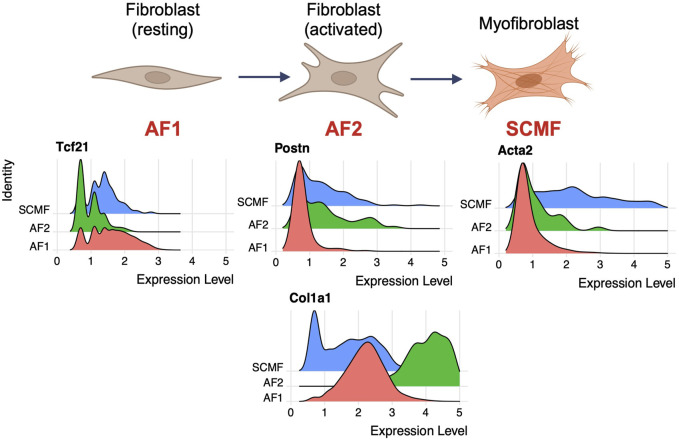
Type 1 collagen is most highly expressed in activated fibroblast cells. Top: Illustration showing different stages of fibroblast activation and differentiation. Bottom: Ridgeplots showing expression of known fibroblast specific markers. In the mouse lung dataset, AF1 cells show the highest levels of Tcf21, AF2 cells show highest levels of Postn, and SCMF cells show highest levels of Acta2. Col1a1 expression is highest in AF2 cells when compared to AF1 and SCMF.

**FIGURE 5:**
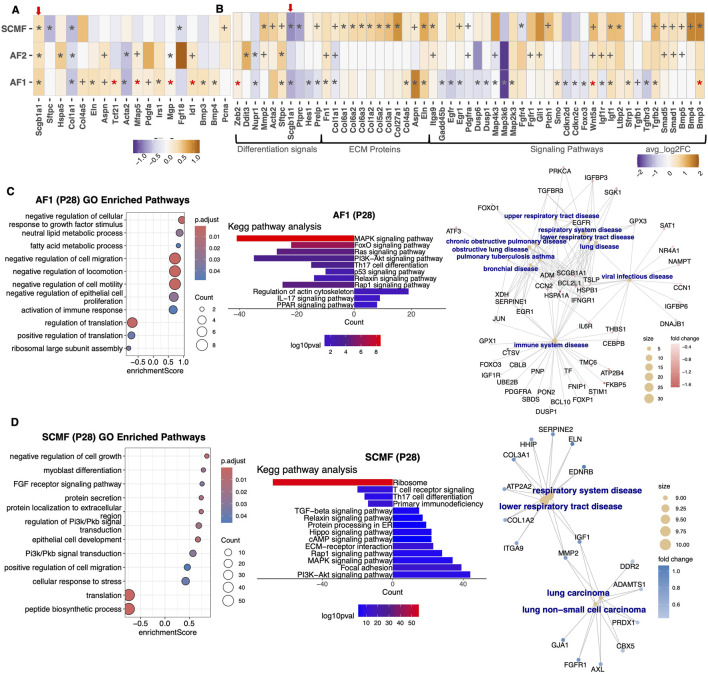
Aga2 fibroblasts show accelerated differentiation and altered ECM expression at p28. **(A)** Heatmap showing differential expression of fibroblast differentiation markers, extracellular matrix expression, and proliferation in Aga2 vs. WT p5 lungs. * = adjusted P value <0.05, red* = adjusted P value <0.05 in some but not all subclusters, + = non-adjust P value <0.05. Red arrow highlights differential expression of Scgb1a1. **(B)** Heatmap showing differential expression of fibroblast differentiation markers, extracellular matrix expression, and signaling pathways in Aga2 vs. WT p28 lungs. * = adjusted P value <0.05, + = non-adjust P value <0.05. Red arrow highlights differential expression of Scgb1a1. **(C)** Left, Middle: Top relevant enriched GO and KEGG pathway analysis terms in differential expression between WT and Aga2 in p28 AF1 fibroblasts. Right: CNET plot of top relevant enriched human diseases in differential expression between WT and Aga2 in p28 AF1 fibroblasts. **(D)** Left, Middle: Top relevant enriched GO and KEGG pathway analysis terms in differential expression between WT and Aga2 in p28 SCMF fibroblasts. Right: CNET plot of top relevant enriched human diseases in differential expression between WT and Aga2 in p28 SCMF fibroblasts.

Using expression data, lung fibroblast types and subtypes were delineated. During homeostasis or following injury, alveolar fibroblasts undergo differentiation from AF1 cells expressing high levels of Tcf21 and Pdgfrα, to AF2 cells expressing Postn and some collagens, and finally to SCMF cells expressing high levels of Acta2 and multiple collagen types (Figure 4). While all three cell types express type 1 collagen, AF2 (activated fibroblasts) expressed the highest levels in the WT lung (see above). In the Aga2 lung at both p5 and p28, the high Col1a1 expressing cell type AF2 showed increased expression of Hspa5 (encoding BiP) and Ddit3 (encoding CHOP), respectively (Figures 5A,B). Higher expression of these genes suggest ER stress through activation of the ATF6 and PERK arms of the unfolded protein response.

In differential expression analysis at p5, Aga2 AF1 cells showed increased levels of Tcf21, Mfap5, Pdgfa, and Irs1 as well as decreased Acta2 expression, an indication of decreased fibroblast differentiation at this timepoint (Figure 5A, Supplementary File S4). p5 AF2 cells also showed decreased Acta2 along with decreased Col1a1 expression as expected. p5 SCMF cells showed decreased Fgf18 expression, a common marker for myofibroblasts and a possible indication of dedifferentiation and diminished proliferation (Ushakumary et al., 2021).

At p28, AF1 cells were subtyped into alveolar fibroblasts, adventitial fibroblasts, and lipofibroblasts. Aga2 AF1 alveolar fibroblasts had decreased expression of Pdgfrα and increased expression of differentiation factors including Bmp3, Tgfβ2, and Sfrp1 (Mayr et al., 2024) (Figure 5B). A subset of AF1 cells (adventitial fibroblasts also) showed an increased in the differentiation markers Bmp3 and Zeb2 along with an increase in Wnt5a expression. Aspn was also upregulated in multiple AF1 clusters along with Tgfβ2 and promotes Tgfβ induced myofibroblast differentiation (Huang et al., 2022). We saw upregulation of Igf1 in alveolar fibroblasts, which is indicative of increased transition to myofibroblasts (Ushakumary et al., 2021). Aga2 p28 SCMF cells also showed increased Bmp3, Igf1, and Ltbp2 expression, as well as decreased transcription of ribosomal genes, an indication of increased differentiation (Figures 5B,D) (Hayashi et al., 2014).

In performing KEGG and GO term analyses, we found changes in several key processes and genes supporting an increase in fibroblast differentiation at p28 (Supplementary Files S5, S6) Aga2 AF1 cells showed increased Pparγ signaling as well as decreased Rap1, FoxO, MAPK, and Pi3k-Akt signaling pathway enrichment (Figure 5C). Pparγ signaling induces fibroblast-to-myofibroblast differentiation (Reddy et al., 2014). Decreased Rap1 signaling induces fibroblast proliferation while decreased FoxO signaling increases myofibroblast differentiation (Luo et al., 2020; Al-Tamari et al., 2018). FoxO signaling is also downstream of Pi3k-AKT activation and both are decreased in Aga2 AFI cells. Further, there was decreased Foxo3, Cdkn2c and Cdkn2d expression nuclear targets of FoxO signaling. Athough Cdkn2c and Cdkn2d are in a class of proliferation inhibitors, their decreased expression has been shown to induce fibroblast differentiation again supporting increased fibroblast differentiation (Scruggs et al., 2018). The decrease in MAPK signaling is likely a decrease in the MAPK/ERK pathway of activity, confirmed by decreased levels of the nuclear targets, Egr1 and Creb3 expression, also indicating alterations in Aga2 AF1 proliferation/differentiation (Wu et al., 2009). Lower activation of MAPK/ERK signaling through Jun, also reduced in Aga2 AF1 fibroblasts, has been seen in fibroblasts derived from COPD patients (Mercer and D'Armiento, 2006). We do, however, see increased Mkk3 (Map2k3) expression in AF1 cells, which is indicative of increased p38/MAPK signaling, as well as the downregulation of their inhibitors Dusp1 and Dusp6. These inhibitors of p38 activation are induced by Tgfβ2 and are shown to increase alveolar fibroblast differentiation (Fortier et al., 2024; Zarubin and Han, 2005). In SCMF Aga2 p28 cells, KEGG analyses supported increased MAPK signaling enrichment, as well as increased Pi3k-Akt, Hippo, Tgfβ, and Rap1 signaling pathways, essentially the opposite pattern of what was seen AF1 cells (Figure 5D). Increased MAPK activation of p38 can lead to increased lung inflammation as well as cell proliferation and differentiation (Garcia-Ryde et al., 2023). In the Aga2 SCMF cells, there was increased expression of Map4k3, a protein kinase involved in mTOR signaling. These findings of upregulation of the p38/MAPK and MTOR pathways may contribute to increased pulmonary inflammation (Yan and Lamb, 2011). Although we did not see any changes in absolute fibroblast numbers, the findings demonstrated an increased differentiation potential in Aga2 fibroblasts. In a human disease analysis (Yu et al., 2015) of p28 Aga2 AF1 and SCMF cells, there were many differentially expressed genes associated with multiple previously shown lung and immune system diseases including COPD, asthma, and respiratory tract diseases (Figures 5C,D).

In addition to dysregulated differentiation gene expression in fibroblasts, the representation of collagens was altered by the Aga2 mutation particularly at the p28 timepoint. The Aga2 AF1 fibroblasts showed an increase in Col4a5 expression and SCMF cells showed increased expression of Col27a1, Col3a1, Col5a2, Col6a1, Col6a2, and Col6a3 (Figure 5B). Similar to AF1 fibroblasts, Col1a2 was increased in SCMF cells, suggesting a possible compensatory mechanism for the decrease in Col1a1 expression. Overall, these findings indicate decreased fibroblast differentiation at p5 and increased differentiation at p28 along with an increase in the expression of extracellular collagens, which could influence the biomechanical properties of the Aga2 lung.

### Aga2 lungs show alterations in epithelial cell differentiation and communication with fibroblasts

**FIGURE 6:**
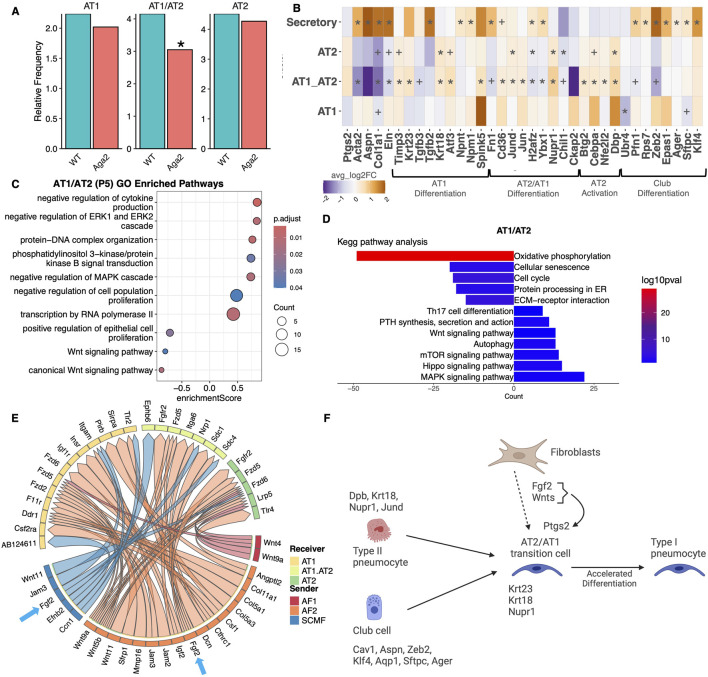
Aga2 epithelial cells show accelerated differentiation in p5 lungs. **(A)** Cell frequency analysis of epithelial cells in p5 WT and Aga2 lungs. * = P value <0.05 via T-test. **(B)** Heatmap showing differential expression of epithelial differentiation markers and extracellular matrix expression in p5 lungs. * = adjusted P value <0.05, + = non-adjust P value <0.05. **(C,D)** Top relevant enriched GO and KEGG pathway analysis terms in differential expression between WT and Aga2 in p5 AT1/AT2 epithelial cells. **(E)** Circos plot showing inferred upregulated in Aga2 cell communication via MultiNicheNet analysis. Receiver epithelial cells with increased expression of signaling receptors and downstream targets are connected to the fibroblast sender cell types expressing ligands predicted to promote this response. Ligands expressed by the same cell population are colored the same. Blue arrows highlight the increased communication via Fgf2. **(F)** Illustration showing accelerated differentiation of epithelial cells in Aga2 p5 lungs via increased expression of differentiation markers and signals from fibroblasts.

Because collagen expressing fibroblasts are so important for the maintenance, proliferation, and differentiation of AT2 and AT1 epithelial cells, we looked for alterations in epithelial cell numbers by performing a cell frequency analysis. We found that at p5, Aga2 lungs showed a significant reduction in the number of AT2/AT1 transition cells but no change in AT1 or AT2 cell numbers (Figure 6A).

We performed differential expression analysis between WT and Aga2 on p5 AT1, AT2, AT2/AT1, and club cell clusters (Figure 6B). At p5, we found that in AT2 cells, there was a significant increase in the expression of Dbp, Krt18, Nupr1, and Jund, genes that are active during the differentiation of AT2 to AT1cells (Strunz et al., 2020). The AT2/AT1 cell cluster showed increased expression of Nupr1, Atf3, Krt23, Krt18, Cebpa, Timp3, CD36, Jund, and Nfe2l2, all genes that are also active during AT2 to AT1 differentiation (Strunz et al., 2020). Club cells also showed increased expression of epithelial differentiation genes including Zeb2, Klf4, and Epas1 as well as Sftpc and Ager indicating increased differentiation potential. GO analysis showed that AT2 cells had significant increases in metabolic activity and KEGG analysis showed decreased oxidative phosphorylation, cell cycle, and DNA replications, an indication of increased differentiation (Supplementary Figures S5A,B). GO analysis of AT2/AT1 cells showed significant increases in positive regulation of RNA transcription but a downregulation of protein translation (Figure 6C). KEGG analysis also showed increased enrichment of MAPK, Hippo, Wnt, and PTH signaling in AT2/AT1 cells, pathways associated with increased epithelial differentiation (Abdelwahab et al., 2019; Volckaert et al., 2019; Liu et al., 2016; Hastings, 2004) (Figure 6D). These data indicate that at p5, the Aga2 AT2 and AT2/AT1 transition cells are demonstrating accelerated differentiation and could therefore deplete the pool of transitioning cells.

The AT2 progenitor cell niche is generally understood to be maintained by signals from matrix fibroblasts. RNASCOPE results showed that Col1a1 expressing fibroblasts are in close proximity to epithelial AT2 and AT1 cells (Supplementary Figure S5C). Communication between these cell types is well-established, therefore, we performed MultiNicheNet analysis to determine whether there were alterations in fibroblast-epithelial communication in p5 Aga2 lungs (Figure 6E; Supplementary Figure S5C, Supplementary File S7). FGF2 expression was increased in AF2 and SCMF cells along with the expression of one its targets, receptor Nupr1, in AT2/AT1 cells. Nupr1 is involved in cellular stress responsiveness and highly active during AT2 to AT1 differentiation supporting previous findings of accelerated epithelial differentiation in Aga2 lungs (Strunz et al., 2020). We saw increased Ptgs2 expression, another target of Fgf2 signaling activation and an activator of macrophages, that is elevated in COPD patients (Duan et al., 2024). The Nichenet analysis also revealed that Ckap2 expression, a gene known to induce proliferation, was reduced in AT1/AT2 cells further supporting increased differentiation (Yoo et al., 2017). Fgf2 ligand expression from AF2 cells was also associated with reduced Chil1 expression, a widely used marker for AT2 cells, and important for their maintenance and function. These results and their effect on differentiation are summarized in Figure 6F.

**FIGURE 7:**
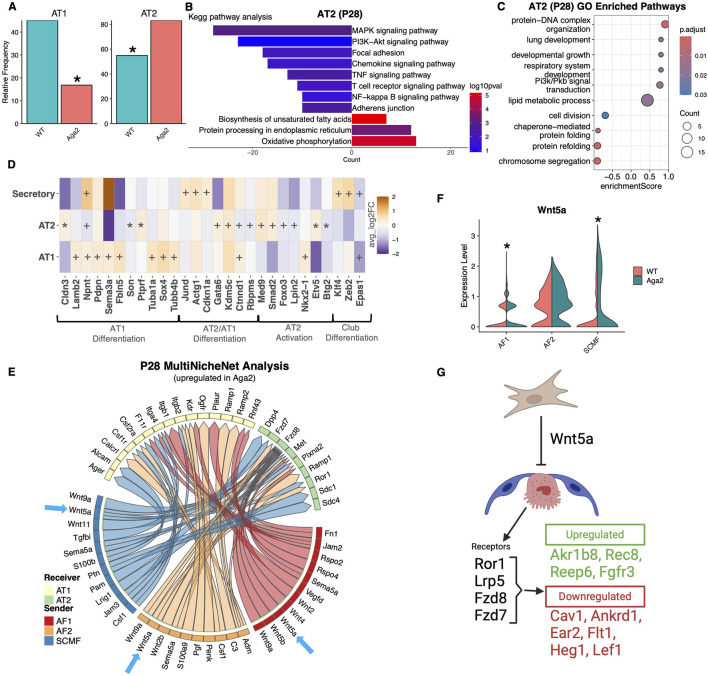
Aga2 p28 AT2 cell differentiation is blocked by Wnt5a signals from fibroblasts. **(A)** Cell frequency analysis of epithelial cells in p28 WT and Aga2 lungs. * = P value <0.05 via T-test. **(B,C)** Top relevant enriched GO and KEGG pathway analysis terms in differential expression between WT and Aga2 in p28 AT2 epithelial cells. **(D)** Heatmap showing differential expression of epithelial differentiation markers p28 lungs. * = adjusted P value <0.05, + = non-adjust P value <0.05. **(F)** Violin plot showing increased expression of Wnt5a in Aga2 fibroblasts. **(E)** Circos plot showing inferred upregulated in Aga2 cell communication via MultiNicheNet analysis. Receiver epithelial cells with increased expression of signaling receptors and downstream targets are connected to the fibroblast sender cell types expressing ligands predicted to promote this response. Ligands expressed by the same cell population are colored the same. Blue arrows highlight the increased communication via Wnt5a. **(F)** Illustration showing blocked differentiation of epithelial cells in Aga2 p28 lungs via increased expression of Wnt5a signals from fibroblasts.

At p28, Aga2 lungs showed a significant increase in the number of AT2 cells and a decrease in AT1 cells (Figure 7A). The increase in AT2 cells was confirmed via *in vivo* quantification of sections labeled with RNAScope probes for SFTPC and AGER (Supplementary Figure S6D). Aga2 AT2 cell GO and KEGG enrichment analysis showed increased fatty acid metabolism, lipid metabolic processing, and decreased metabolic activity specifically oxidative phosphorylation and aerobic respiration indicating a quiescent phenotype (Figures 7B,C). We also saw a decrease in MAPK signaling activation, which when reduced inhibits AT2 cell differentiation (Liu et al., 2016). When AT2 cells were interrogated for differentiation markers, we found decreased expression of Son and Btg2, both normally upregulated during differentiation to AT1. There was increased expression of Cldn3, a tight junction protein that is normally decreased during epithelial cell transdifferentiation (Chen et al., 1985) (Figure 7D). With the increase in AT2 cell numbers in Aga2 p28 lungs, these data suggest that AT2 cells are prevented from differentiating and therefore deplete the available AT1 pool of cells.

Our fibroblast/epithelial MultiNicheNet analysis revealed increases in Wnt, Tgfβ/BMP, and FGF signaling (Figure 7E; Supplementary Figures S6A,B, Supplementary File S8). Wnt5a is important in maintaining the AT2 cell niche and preventing differentiation into AT1 cells. At p28, Wnt5a expression was increased in AF1 matrix fibroblasts and as were the downstream Wnt targets in AT2 cells that include Akr1b8 (essential for proliferation (Shen et al., 2015), plays a pro-inflammatory role (Rajak et al., 2022), upregulated in mouse COPD model (Gao et al., 2022)), Fgfr3 (increases epithelial proliferation (Yin et al., 2016)), Rec8 (necessary for meiosis (Sakuno et al., 2022)), and Reep6 (increases inflammation through Cxcr1 (Park et al., 2016)) (Figures 7F,G). Additional factors in fibroblast-epithelial communication were also increased including Mdk, Wnt4, and Wnt5b, ligands that are associated with increased expression in COPD (Deng et al., 2022; Wu et al., 2019; Durham et al., 2013). These results and their effect on differentiation are summarized in Figure 7G.

### Immune cells in Aga2 lungs show altered frequency, expression, and communication with fibroblasts and epithelial cells

**FIGURE 8:**
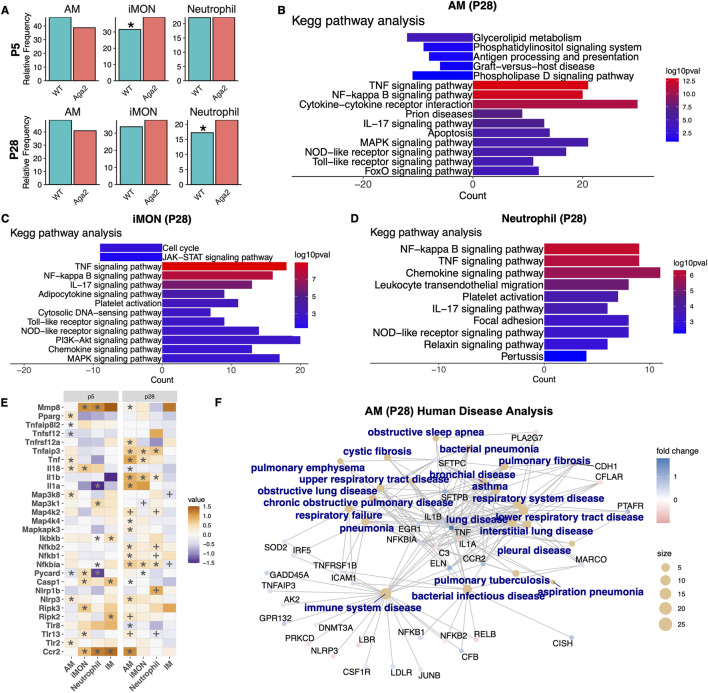
Aga2 lungs show increased immune cells and inflammation via NOD-like Receptor pathway activation. **(A)** Cell frequency analysis of immune cells in p28 and p5 WT and Aga2 lungs. * = P value <0.05 via T-test. **(B–D)** Top relevant enriched KEGG pathway analysis terms in differential expression between WT and Aga2 in p28 AM, iMON, and neutrophil cells. **(E)** Heatmap showing differential expression of inflammation and NOD-like receptor signaling pathway markers in p5 and p28 lung immune cells. * = adjusted P value <0.05, + = non-adjust P value <0.05. **(F)** CNET plot of top relevant enriched human diseases in differential expression between WT and Aga2 in p28 alveolar macrophages.

Alveolar macrophages and associated immune cells for example, iMONs and neutrophils play key roles in the progression of emphysema and COPD (Wu et al., 2022). *Theile et al.* observed an infiltration of neutrophils and alveolar macrophages in the lungs of Aga2 mice. Therefore, we performed a cell frequency analysis and found a significantly increased number of inflammatory monocytes in p5 and p28 Aga2 lungs as well as increased neutrophil numbers at p28 indicating an increase in overall inflammation supporting previously reported findings (Figure 8A).

Differential expression analysis at p5 showed increased Ccr2 expression in Aga2 inflammatory monocytes, neutrophils, and interstitial macrophages, which causes chronic lung inflammation through the recruitment and activation of monocytes (Dong et al., 2024). GO term analysis showed increased fatty acid catabolism, DNA replication, and RNA transcription in AMs, an indication of increased replication (Supplementary Figure S7A). iMONs showed enrichment of genes associated with the immune response, cell migration, and epithelial cell development (Supplementary Figure S7B). p5 neutrophils showed decreased ribosomal gene expression and cell cycle, an indication of increased cellular differentiation (Supplementary Figure S7E). KEGG pathway analysis in p5 AMs, iMONs, and neutrophils showed increased enrichment of the NOD-like receptor (NLR) signaling pathway (Supplementary Figures S7C–E). The NLR pathway activates the early innate inflammatory response mainly through Nfκβ or MAPK signaling, which then induce expression of inflammatory cytokines like Il1b, Il18, and Tnfs (Almeida-da-Silva et al., 2023). The NLR pathway can be activated via Toll-like receptors, and we found increased expression of Tlr2 and Tlr13 in Aga2 p5 AMs and iMONs, respectively (Figure 8E). There was increased Ripk2 and Ripk3 expression in iMONs, the main effectors of NLR signaling that activate MAPK and Nfκβ signaling (Schenten and Medzhitov, 2011) (Figure 8E). KEGG analysis showed decreased enrichment of MAPK signaling and increased Nfκβ signaling (Supplementary Figure S7D). Specifically, neutrophils showed increased levels of Nfkbia and Ibkb, both effectors of Nfκβ signaling (Liu et al., 2017) (Figure 8E). Looking downstream, there was increased expression of Il18 in p5 inflammatory monocytes and AMs (Figure 8E). Il18 is a nuclear target of NLR signaling and is also a driver of inflammatory cell accumulation that has been linked to the development of emphysema (Hoshino et al., 2007). p5 Aga2 neutrophils, IMs, AMs, and iMONs all showed increased expression of Tnf, Tnfrsf1b, Tnfsf12, Tnfalp8l2 supporting NOD-like receptor pathway enrichment (Figure 8E). Further, p5 Club cells showed increased expression of Cldn5, a tight junction protein induced by TNF inflammatory signaling that could result in disruption of the epithelial cell barrier and leakage of fluids into the surrounding tissue, increasing inflammation (Wittekindt, 2017) (Figure 6B). The genes Casp1 and Pycard were also upregulated along with Nlrp3 in Aga2 immune cells (Figure 8E). Proteins from these genes comprise the inflammasome complex whose dysregulation can cause chronic inflammation (Zheng et al., 2020). Human disease analysis on p5 immune cells showed genes associated with lung and immune system diseases such as pneumonia, rheumatoid arthritis, respiratory distress syndrome, and autoimmune disease (Supplementary Figure S7E). Overall, at p5, Aga2 lungs show an increase in early inflammatory signals through the NOD-like receptor/Nfκβ pathways when compared to WT, which could be affecting early postnatal lung development.

At p28, GO analysis revealed increased inflammatory response and neutrophil chemotaxis in Aga2 AMs as well as cell migration and cytokine production in Aga2 iMONs, again supporting the increased frequency of inflammatory cells in the Aga2 lung (Supplementary Figures S10A,B). KEGG and GO term analyses showed that Aga2 AMs, iMONs, and neutrophils had increased NLR, Nfκβ, MAPK, and Tnf pathway activation, pro-inflammatory pathways implicated in emphysema and COPD (Liu et al., 2017) (Figures 8B–D; Supplementary Figures S10A,B). Differential expression analysis of alveolar macrophages showed increased Il1α, Il1β, and Ccr2 expression in Aga2, all proinflammatory factors (Figure 8E). Il1β expression was also increased in both inflammatory monocytes and neutrophils and is a pro-inflammatory mediator in COPD as well as a nuclear target of NLR signaling (King, 2015; Cosio et al., 2009) (Figure 8E). We saw the increased expression of Tnf, Tnfaip3, and Tnfrsf12a in Aga2 p28 AMs, iMONs, and neutrophils (Figure 8E). We also saw increased expression of Nfkbia and Nfkb1 as well as several mediators of MAPK signaling (Figure 8E). This indicates that at p28, increased NLR signaling in immune cells could be inducing Il and Tnf chemokine expression through both MAPK and Nfκβ pathways. Pparγ expression was increased in Aga2 AMs, which is involved in activating alveolar macrophage activity to dampen inflammatory responses, thus attempting to resolve the inflammatory phenotype (Asada et al., 2004) (Figure 8E). There was also increased Mmp8 expression in Aga2 AMs, which cleaves collagens and releases matrikines that enhance neutrophil recruitment (Kulkarni et al., 2016) (Figure 8E). Human disease analysis of AM and iMON differential expression showed the association of many differentially expressed genes with diseases characterized by chronic inflammation such as emphysema, cystic fibrosis, asthma, pulmonary fibrosis, COPD, pneumonia, and respiratory failure (Figure 8F; Supplementary Figure S10C). This analysis shows that at p28, the inflammatory phenotype is more pronounced in Aga2 than at p5, showing increased inflammatory signaling pathways as well as inflammatory cell recruitment and activation.

**FIGURE 9:**
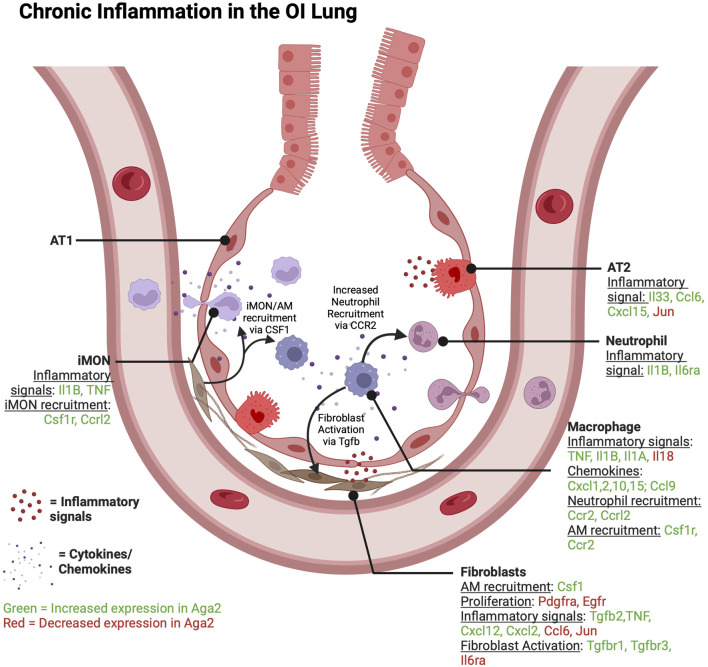
Aga2 lungs show a chronic inflammatory phenotype. Illustration showing increased and decreased markers of inflammation and how Aga2 mouse lungs are an example of chronic inflammation via increased TNF and Il1B signaling activation and recruitment of macrophages and neutrophils into the alveolar space. Immune cells may also play a role in increasing fibroblast activation via the expression of Tgfβ ligand.

Communication between fibroblasts, epithelial cells, and alveolar macrophages is established and plays an important role in lung homeostasis (Bissonnette et al., 2020). This communication influences both macrophage recruitment as well as fibroblast activation/proliferation and epithelial cell differentiation. Because of altered expression in Aga2 immune cells, we performed MultiNicheNet analysis to determine whether there were changes in cell-cell communication between epithelial and immune cells (Figure 9). At p5, we saw an increase in Csf2 ligand expression from AT1/AT2 and AT2 cells and increased Csf2ra receptor expression in IMs and neutrophils (Supplementary Figure S8). Similarly, at p28, we saw an increase in Csf1 and Csf2 ligand expression coming from AT1 and AT2 cells, respectively (Supplementary Figure S11). We also observed an increase in Csf receptor expression in AM cells (namely, Csf1r, Csf2ra, and Csf3r) (Supplementary Figure S13). The expression of Csf by epithelial cells plays an important role in triggering the recruitment of alveolar macrophages in response to infection (Bissonnette et al., 2020). However, an exaggerated and perhaps chronic response could result in severe lung inflammation through the influx of inflammatory monocytes and neutrophils, seen in our single cell data. At p28, we also observed an increase in Il33 expression in Aga2 AT2 cells along with increased expression of its targets, Cdh1, Ptgs2, and Ms4a7, in AM cells (Supplementary Figure S13). Il33 is a well-known promoter of immunity and immune cell recruitment (Chapman, 2021). Wnt, TNF, and Tgfb signals from immune cells were increased in Aga2 and their downstream targets Reep6, Cxcl2, and Ccn2, respectively, were increased in Aga2 epithelial cells (Supplementary Figures S11, S13).

We then analyzed fibroblast to AM cell communication. At p5, we saw increased activation of the Tlr2 receptor with Ccn1 signals coming from SCMFs to neutrophils and IMs as well as from AF2s to AMs (Supplementary Figure S9). Ccn1 signals from the same cells were also paired with Itga5, Itgb2, and Itgam activation in AMs. We saw similar patterns at p28 where Ccn1 expression is upregulated in AF1 cells and indicate increased Itga5, Itgb2, and Itgam activation (Supplementary Figures S12, S13). Ccn1 is known to bind to Tlr2, activate inflammation through Il1B and Tnf, and recruit monocytes (Jun and Lau, 2020; Bai et al., 2010). Ccn1 expression is also highly influenced by mechanical stretch of the ECM, particularly in the lung (Morrell et al., 2020). This could point to increased Ccn1 expression in Aga2 fibroblasts as one of the instigators of the chronic inflammation phenotype. We also saw increased Csf1 ligand expression coming from p28 AF1 and AF2 cells, leading to increased expression of the nuclear targets Csf1r, Egr1, Il1b, Jun, and Tnf in Aga2 AMs, all factors involved in increased AM recruitment and overall inflammation (Luca et al., 2021; Garon et al., 2020; Herold et al., 2006; Srivastava et al., 2005; Gordon, 2003) (Supplementary Figures S12, S13). The nuclear target Id1, a gene important to the anti-inflammatory response was decreased in Aga2 AMs (He et al., 2023) (Supplementary Figure S13). When we analyzed the communication of AMs to fibroblasts, we saw an increase in Tgfβ1 expression in AMs and a subsequent increase in the target genes Col1a2, Fzd8, Mmp2, Mrc2, Plod2, Serpine2, Sox4 and Adamts6 in Aga2 AF cells (Supplementary Figure S13). We also saw increased Il1b and Il1a signals from Aga2 AMs resulting in increased expression of target genes Il1b, Tram2, and Ptger4 involved in inflammation, type 1 collagen synthesis, and fibroblast differentiation (Bozyk and Moore, 2011; Stefanovic et al., 2004). Additionally, we observed decreased levels of Pdgfrα and Egfr receptors in AF cells (Figure 5B). These signaling pathways play major roles in AM-Fibroblast communication. Decreased Pdgf and Egf signaling in AFs results in decreased fibroblast proliferation while increased Tgfβ and Il signaling from AMs results in increased fibroblast activation (reviewed in Buechler et al. (2021)) Overall, these data indicate alterations in alveolar macrophage gene expression and communication in Aga2 lungs leading to increased inflammation and inflammatory cell recruitment as well as increased fibroblast activation.

### Scgb1a1 expression is differentially expressed in multiple lung cell types

Scgb1a1 is a secretoglobin also known as club cell secretory protein (Ccsp) that is highly expressed in and commonly used as a marker for distal airway club cells. Recently, it has been shown to be expressed in other lung cell types including immune cells and fibroblasts. In general, Scgb1a1 is known to have anti-inflammatory effects, and reduced levels are seen in COPD and asthma (Martinu et al., 2023).

In our single cell data, we found increased levels of Scgb1a1 in p5 AMs and fibroblasts (red arrows in Figure 5A; Supplementary Figure S13) along with decreased AM expression of Socs-3 (a nuclear target inhibited by Scgb1a1 expression) (Ray et al., 2006). At p28, however, alveolar macrophages as well as neutrophils and T cells showed significantly decreased Scgb1a1 expression along with increased expression of Socs-3 and Ptgs2 in AMs (nuclear targets inhibited by Scgb1a1 expression) (Ray et al., 2006; Mandal et al., 2004). We also saw decreased Scgb1a1 expression in Aga2 p28 fibroblasts, AT1, and Club cells (red arrows in Figure 5B; Supplementary Figure S13). Reduced expression of Scgb1a1 results in early expression of inflammatory pathways and increased release of cytokines and chemokines including Il1b and Tnfα associated with obstructive lung disease (Xu et al., 2020). This data is consistent with the increased inflammation specifically through Il1b and Tnf signaling seen in Aga2 AM cells in our single cell analysis. These results indicate that inflammation and the reduction of Scgb1a1 could play a role in why OI patients have lung insufficiency and do worse in the face of lung damage.

## Discussion

Pulmonary complications significantly cause mortality in OI and studies show phenotypic heterogeneity with more severe outcomes associated with the severe progressive deforming (type III) and moderately affected (type IV) forms of OI, respectively. Even patients with mild OI (type I) show decreased pulmonary function and complications, with increased mortality even in the absence of spinal and rib deformations, the same trend as observed in more severe forms of OI (Thiele et al., 2012). Multiple OI mouse models display intrinsic pulmonary abnormalities. The *Col1a1* ^*Jrt/+*^ mouse displayed alveolar hypoplasia and the *Crtap* ^*−/−*^ mouse showed functional lung abnormalities that resembled both restrictive and obstructive disease that could be partially rescued with anti-TGFβ treatment (Baglole et al., 2018; Bald et al., 2010) Aga2 mice exhibit a range of phenotypic severity. Studies of the severe form, but not milder forms of the Aga2 mouse, uncovered diffuse pulmonary hemorrhage and inflammation along with disorganized ECM and respiratory failure (Thiele et al., 2012). The difference between the moderate versus severe phenotype in Aga2 mice is poorly understood and the severe phenotype was rarely seen in our colony. Therefore, we only studied the lungs of moderately effected Aga2 mice to report on a population that is more representative of the Aga2 mouse model. In this study, we found increased alveolar spacing in Aga2 lungs at postnatal day p28 resulting in an emphysematous phenotype. By performing scRNAseq analysis, we were able to show that the Aga2 lung exhibited alterations in fibroblast and epithelial cell differentiation, altered ECM component expression, as well as an increase in inflammation and inflammatory cell recruitment, a hallmark of the lung diseases emphysema, COPD, and cystic fibrosis.

The interstitial ECM of alveoli is composed mainly of type I and type III collagens, which provide the hysteresis and viscoelasticity necessary for respiration as well as governing cell-matrix adhesions through interactions with integrins (Frantz et al., 2010; Bosman and Stamenkovic, 2003). The behavior of lung cell types that include fibroblasts, macrophages, endothelial, and epithelial cells change based on matrix stiffness in both diseased and normal lung tissues (reviewed in Burgstaller et al. (2017)). Further, compositional changes in the lung ECM have been tied to several congenital and progressive diseases such as idiopathic pulmonary fibrosis (IPF), chronic obstructive pulmonary disease (COPD), pulmonary arterial hypertension (PAH), and asthma (reviewed in Burgstaller et al. (2017)) many of which show increased expression of ECM components. In this study, Aga2 fibroblasts had significantly decreased expression of type 1 collagen. These data complement our earlier findings in Aga2 cartilage as well as *Thiele et al.* showing decreased RNA expression specifically of the mutant Col1a1 allele in Aga2 mouse heart and lung tissues. However, because lung cells express low levels of type 1 collagen, we may also more easily see increased Col1a1 expression in certain cell types as a compensatory mechanism for the overall decrease in Col1a1 protein. But fibroblasts also showed increased expression of type III, IV, V, VI, and XXVII collagens, as well as Fibrillin-1 (Fn1) and Elastin (Eln), other established ECM proteins. These changes in expression likely lead to tissue-wide effects on cell function as well as overall lung biomechanics.

Within the lung interstitium, fibroblasts are the most commonly found cell type and responsible for ECM secretion during lung development and homeostasis while also serving as effector cells during injury or repair (reviewed in Mammoto and Mammoto (2019)) They also play a major role in epithelial cell development and renewal. During early postnatal development (p5) of the Aga2 lung, we found overall that Aga2 fibroblasts displayed a quiescent cell state with a reduction in metabolism and reduced differentiation. However at p5, epithelial cells were altered with a significant decrease in the number of AT2/AT1 transition epithelial cells as well as increased expression of epithelial cell differentiation markers in club, AT2, and AT2/AT1 cells along with increased MAPK signaling enrichment, implying increased differentiation. Though we do not see an emphysemous phenotype in the developing lung at p5, our study shows altered fibroblast and epithelial development as well as early signs of inflammation in the Aga2 lung that could lead to changes we see in the mature lung.

In the mature Aga2 OI lung at p28, we saw an increase in fibroblast activation and differentiation to myofibroblasts. AF1 cells at p28 showed decreased expression of several signaling pathways whose downregulation is important to fibroblast activation including MAPK, FoxO, Pi3k-AKT, and Rap1, suggesting accelerated differentiation. Conversely, we saw increased enrichment of these same pathways along with Hippo and Tgfβ signaling in Aga2 SCMFs, which could result in increased inflammation. Interestingly, we did not see major expression or pathway activation changes in AF2 cells even though they are the highest expressors of type I collagen. We did, however, see indications of increased ER stress suggesting that protein misfolding may be playing the largest role in the Aga2 AF2 phenotype. Overall, the major pathways involved in alveolar fibroblast differentiation were increased in Aga2 lungs at p28 are likely due to a cascade of signaling alterations caused by ECM alterations and the expression of both decreased and mutant type I collagen. With their importance in ECM secretion, the accelerated differentiation of Aga2 fibroblasts could affect overall lung structure or development.

At p28, we saw increased numbers of AT2 cells and decreased AT1 cells. Aga2 AT2 cells also show decreased markers of differentiation. Fibroblasts physically interact with AT2 and support capillary structures through the remodeling of local ECM components, thereby supplying growth factors necessary to maintain alveolar homeostasis (reviewed in Mammoto and Mammoto (2019)). In our study, we saw increased Wnt5a expression from Aga2 p28 AF1 fibroblasts and using MultiNicheNet analysis, showed that Wnt5a activation of WNT signaling also occurs in AT2 cells. Wnt5a expression by alveolar fibroblasts has been shown to prevent AT2 to AT1 cell differentiation. Increased expression of Wnt5a by AFI cells may be contributing to this altered mechanism. This impaired AT2 to AT1 transition was described in a mouse model of COPD that exhibited increased alveolar spacing (Yu et al., 2022). This study also saw a decrease in MAPK signaling activation in AT2 cells following COPD induction (Yu et al., 2022). A previous study showed that increased mechanical tension activated MAPK signaling in AT2 cells and induced alveolar regeneration following pneumonectomy. Whereas with reduced mechanical tension they observed decreased MAPK signaling and AT2 differentiation (Liu et al., 2016). Our analysis shows decreased MAPK signaling in Aga2 AT2 cells. Because it is known that MAPK signaling is modulating by mechanical tension, the decrease in signaling could be the result of a reduction in lung mechanical tension due to decreased type 1 collagen expression and increased elastin expression resulting in reduced AT2 cell differentiation (Burkholder, 2007). Although we saw decreased type I collagen and increased Eln expression at both timepoints, the ECM plays different roles in the developing versus the mature lung. Whereas mechanical stretching from amniotic fluid and air is important to induce epithelial differentiation in the developing alveolar sacs, once the lung structure is established decreased mechanical tension results in decreased epithelial differentiation. Overall, the mature Aga2 lung showed increased fibroblast activation and decreased epithelial differentiation. These two processes play major roles in the resolution of lung damage through the formation of fibrotic tissue to stabilize the damage and renewal of epithelial cells through proliferation and differentiation (Lucas et al., 2020).

*Thiele et al.* performed cDNA-chip-based expression profiling of whole lungs from p11 *Aga2* and WT mice and discovered dysregulation of 149 genes (Thiele et al., 2012). The results showed increased ECM-related transcript expression, markers for inflammation and wound-healing, as well as hypoxia. Our single cell analysis also revealed increased expression of ECM related transcripts, inflammatory markers, increased numbers of multiple inflammatory cell types, and increased expression of genes in fibroblast and epithelial cells known to recruit and activate inflammatory cells. Inflammation in the OI lung is, in mice or humans, understudied. Chronic inflammation is well studied in more common diseases like COPD and cystic fibrosis, and both the developing and mature Aga2 OI lung showed many of the same characteristics as other mouse models of these diseases (Oz et al., 2022; Yu et al., 2022; Xu et al., 2024; Petrocheilou et al., 2022). Increased neutrophil numbers and Ccr2 expression are hallmarks of cystic fibrosis, which was seen in our Aga2 lungs (Oz et al., 2022). The Aga2 lungs showed increased communication between AMs and alveolar fibroblasts using Tnfsf12 and Tnfrsf12a, respectively, which promotes inflammation, fibroblast proliferation, and secretion of collagen as seen in COVID-19 (Guo et al., 2024). Increased Toll-like receptor expression is closely associated with cystic fibrosis, COPD, and asthma (Arora et al., 2019). NLR signaling downstream of TLR signaling is known as a ‘master regulator’ of inflammation and is also closely associated with the inflammatory lung phenotypes COPD, pneumonia, and asthma (Saxena and Yeretssian, 2014; Chaput et al., 2013). We further saw increased expression of Pycard, Casp1, and Nlrp3 in Aga2 immune cells, which are downstream of NLR signaling activation suggesting increased inflammasome activation which plays a key role in COPD (Zheng et al., 2020; dos Santos et al., 2012). It is unclear where the chronic inflammation begins, however, the data points to the possibility of increased Ccn1 expression by Aga2 fibroblasts due to altered ECM mechanics affecting integrin signaling and activating of TLR signaling in Aga2 immune cells.

Chronic inflammation could also be affecting epithelial cells in the OI lung. Inflammation can cause damage to tight junctions during homeostasis, increasing permeability and leakage of fluids into the surrounding tissues (Coyne et al., 2003; Carson et al., 1990). This is reflected in our data with the increased expression of Cldn3 in Aga2 p28 AT2 cells, and Cldn5 in Aga2 p5 club cells (Wittekindt, 2017; Wray et al., 2009). Cldn3 and Cldn5 are tight junction claudins expressed in lung airway and alveolar epithelial cells whose upregulation can result in increased epithelial barrier permeabilization. This may allow more cytokines and chemokines from fibroblasts to flow into the alveolar space. Inflamed epithelial cells will also further release pro-inflammatory cytokines including TNFα and Il-1β, amplifying the inflammatory response and recruiting neutrophils and macrophages (Burgoyne et al., 2021; Whitsett and Alenghat, 2015). In what is generally understood concerning inflammatory disease, it is epithelial cell damage that induces an inflammatory state in the lung recruiting immune cells and activating differentiation (Whitsett and Alenghat, 2015). However, it is possible that in Aga2 lungs, inflammatory signals begin from the fibroblasts as a result of abnormal type 1 collagen expression, but this question remains to be explored.

An interesting finding in our data was the differential expression of Scgb1a1 in multiple cell types. At p5 Scgb1a1 expression was increased while at p28 it was decreased. How expression is regulated is not well known although connections with Hippo signaling and Foxa2 expression have been recently made (Zhu et al., 2019; Chung et al., 2013). However, we did not see changes in Foxa2 or Hippo signaling in our data. What is known is that Scgb1a1 plays an anti-inflammatory role in the lung and is downregulated in COPD, cystic fibrosis, and asthma but upregulated in pulmonary fibrosis (Martinu et al., 2023). As a component of pulmonary surfactant and highly expressed in airway Club cells, Scgb1a1 reduces inflammation through the sequestration of prostaglandins and inhibition of NF-κB in the alveolar space (Mootz et al., 2022; Mandal et al., 2005). It is also an inhibitor of Socs-3, which plays a major role in initiating the Th2 immune response and is upregulated in p28 AMs (Ray et al., 2006; Seki et al., 2003). Ptgs2 expression is also inhibited by SCGB1A1 and increased in p28 Aga2 neutrophils (Mandal et al., 2004). PTGS2 is a key enzyme in the synthesis of inflammatory lipid mediators and has been associated with epithelial barrier disruption (Mandal et al., 2004; Park et al., 2007). Why Scgb1a1 is increased in the p5 lung is unclear but could be related to the increased differentiation of Club cells at this timepoint. It is also unclear why Scgb1a1 shows significantly reduced expression in Aga2 p28 lungs. It is possible that the induction of inflammation results in reduced Scgb1a1 expression as has been shown previously, but this mechanism is still tenuous (Lensmar et al., 2000). What is clear is that induction of Scgb1a1 expression has become a promising therapeutic target especially for COPD, cystic fibrosis, and asthma (Martinu et al., 2023; Laucho-Contreras et al., 2016).

In our study we show that Aga2 mouse shows an emphysemous lung phenotype and, using single cell RNA sequencing, we identified alterations in fibroblast and epithelial cell differentiation as well as a chronic inflammatory phenotype. From our data, we were able to elucidate the effect of abnormal type I collagen expression on the Aga2 lung as well as make connections with other inflammatory lung diseases like emphysema, COPD, and cystic fibrosis. In general, the OI lung phenotype does not fully replicate any of the more common lung diseases, implying that understanding the commonality and differences in the OI lung will help guide treatments. We hope that by using modern sequencing technology our study can inform clinical care of OI patients with respiratory insufficiency and prevent early mortality.

## Methods

### Histological analysis/MLI quantification

Paraffin-embedded whole lungs from p28 mice were sectioned at 5 μm and then stained for morphology via Hematoxylin and Eosin staining. MLI quantification was performed using the Fiji/ImageJ package. Eight horizontal and 10 vertical lines of size 745.28 μm and 558.96 μm, respectively, were generated on at least 10 histological fields per mouse lung paraffin embedded lung section H&E images. Intersections were quantified via thresholding and chord measurements. The equation used to determine MLI was *L*_m_ = [*N* horizontally + *N* vertically) × L]/I, where *N* is the number of times the transverse were placed on the tissue, L is length of the transverses, and I is the sum of all intercepts from each field (Dunnill, 1962).

#### ScRNA-seq analysis

Initial data processing was completed using the Cell Ranger pipeline (10x Genomics) for demultiplexing, barcode assignment, and unique molecular identifier quantification. Downstream analyses were performed using Seurat V5. Cells with >6,000 and <200 expressed genes as well as >10% mitochondrial transcripts were excluded (Supplementary Figure S12). Ribosomal genes were not removed and cell cycle correction was not performed. No batch effects were detected and therefore no batch correction was performed. Data normalization for clustering was performed using the SCTransform normalization method. Differential expression profiles derived from normalized RNA counts were analyzed using EnrichR for KEGG pathway analysis, and GSEA for GO and Human Disease analysis. Cell communication analysis was performed using the MultiNichenetr package, which uses the differential expression between conditions and ligand-receptor pairs for prioritization (https://github.com/saeyslab/multinichenetr).

### RNAscope analysis/epithelial cell quantification

RNAscope analysis was performed on whole lung samples fixed in 4% paraformaldehyde overnight followed by paraffin embedding. Lung sections were probed using the RNAscope Multiplex Fluorescent V2 assay from ACDBio. The standard protocol was followed. RNAscope-processed slides were imaged at ×20 original magnification using the Echo Revolution microscope, and images were processed using Adobe Photoshop. Probes used were Col1a1 (catalog 319371), Sftpc (catalog 314101), Ager (catalog 550791), Tcf21 (catalog 508661), Mfap5 (catalog 490211). Three WT and four Aga2 lung samples were separately imaged with the *Echo Revolve* microscope. To optimize the cell counting procedure for DAPI-stained cells, a section size of 214.428 µm × 178.926 µm was selected, roughly one-fourth of an individual field size at 20x for the *Echo Revolve*. To account for small differences in magnification across samples, *Adobe Photoshop* was used to measure scale bars in order to convert section sizes for equal sampling of cells across the lung area. A total of ten sections were randomly chosen from the distal regions of the lung for each sample, and then separately cropped for individual analysis of total cell count (DAPI), AT1 cells (FITC), and AT2 cells (TX RED). *Fiji ImageJ* was used for all the cell quantification. Total cells were quantified first through *ImageJ*’s Particle Analysis function. Images were thresholded at variable intervals to increase contrast between the cells and background before undergoing Dilation and Watershed separation. After thresholding, particle analysis parameters were consistently set at [100-Infinity] pixel units for size and [0.30–1.00] for circularity, generating an image overlay estimating circular regions of cells. Each section was then individually analyzed to add individual cell counts from marked clusters. AT1 and AT2 cells were hand-counted using the *Cell Counter* plugin. Technical replicates were conducted by taking another set of ten random sections from each sample and performing the same analyses for cell counts.

### Statistics

MLI and epithelial cell counts data were acquired using ImageJ (NIH) and analyzed using GraphPad Prism using the Student’s t-test (2 tailed) to determine significance. Data represent mean ± SD. scRNASeq data were analyzed, visualized, and statistically compared using R and the Seurat V5 package. *P* < 0.05 was considered significant.

## Data Availability

scRNA-Seq data sets have been deposited in the National Center for Biotechnology Information’s Gene Expression Omnibus (accession number: GSE304840, https://www.ncbi.nlm.nih.gov/geo/query/acc.cgi?acc=GSE304840). Supporting analytic code can be accessed on GitHub (https://github.com/jenzieba/Cartilage_ScRNAseq; commit ID c79e0b9). All data associated with this study are present in the paper, Supporting Data Values file, Supplementary Datas S1–S8, or public repositories. Please contact the corresponding author for reagents and resources generated in this study.
