# Supplementary figures and images for: Lung scRNA-seq reveals chronic inflammation and emphysemous phenotype in mice with osteogenesis imperfecta
Source: Front Genet. 2026 Feb 26;17:1713393. doi: 10.3389/fgene.2026.1713393 (PMC12978693; doi:10.3389/fgene.2026.1713393)

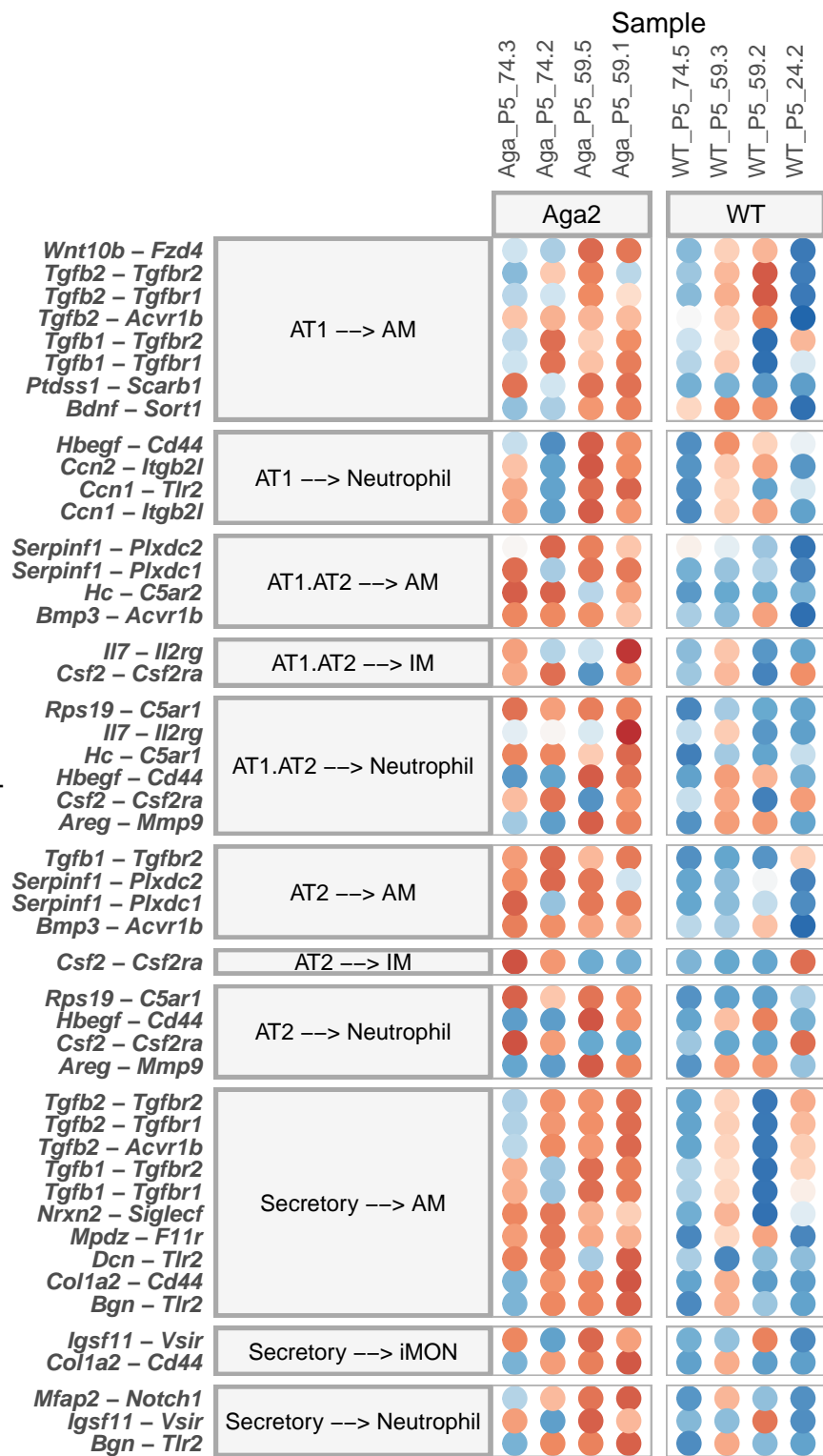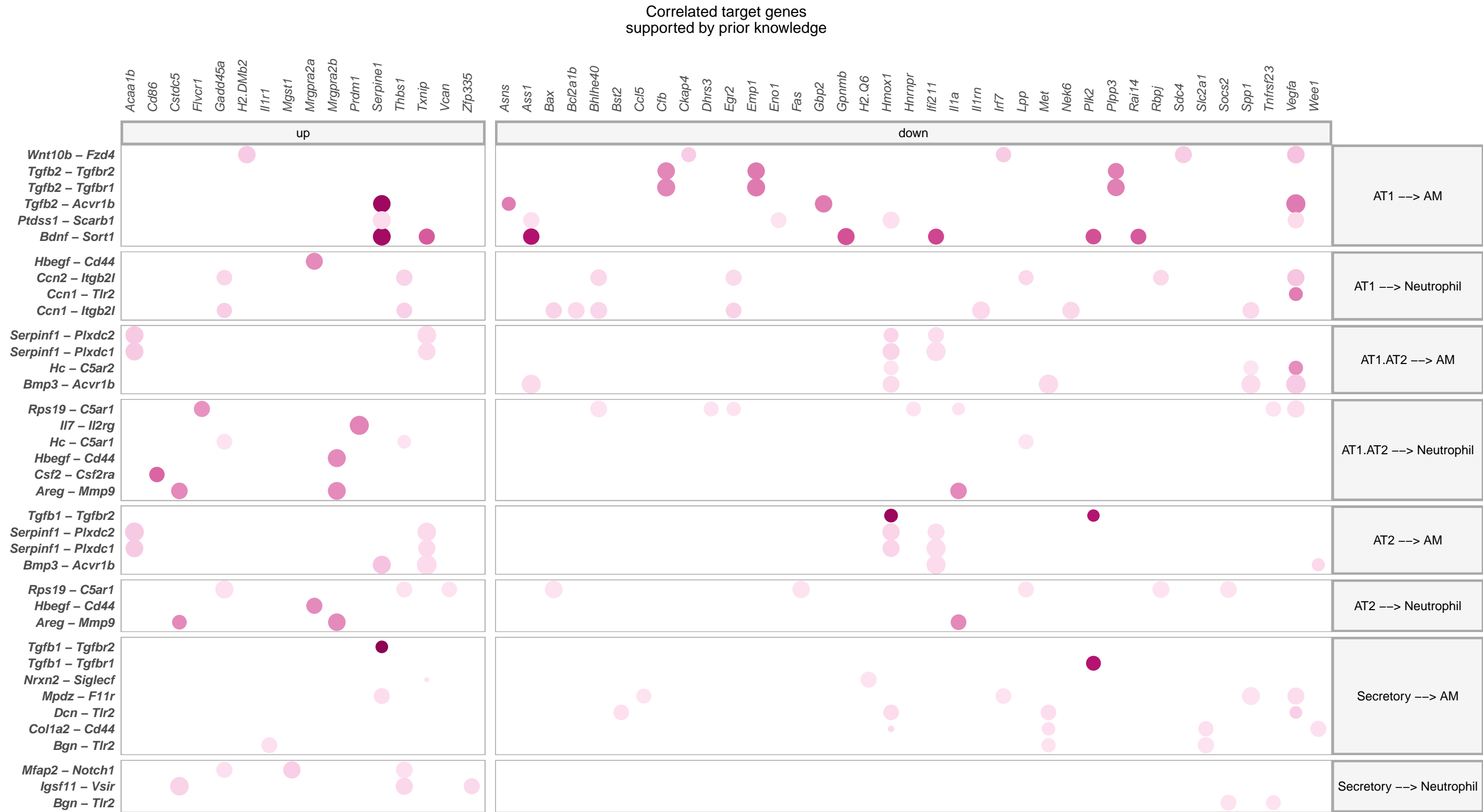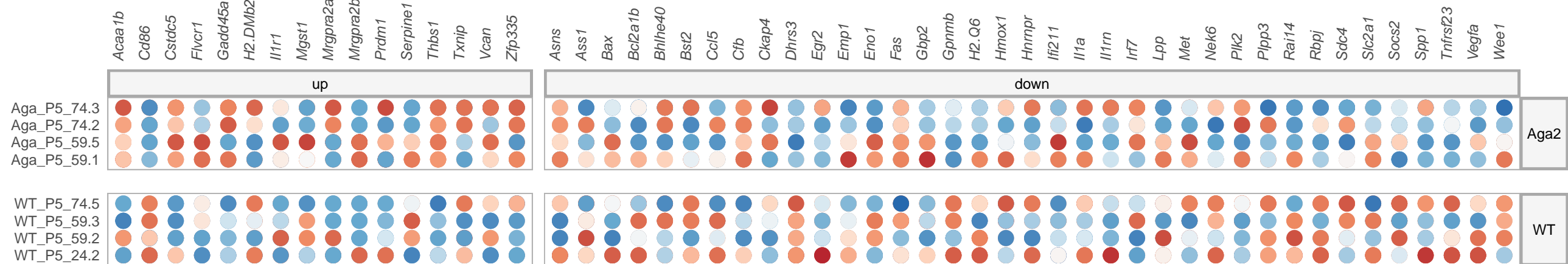

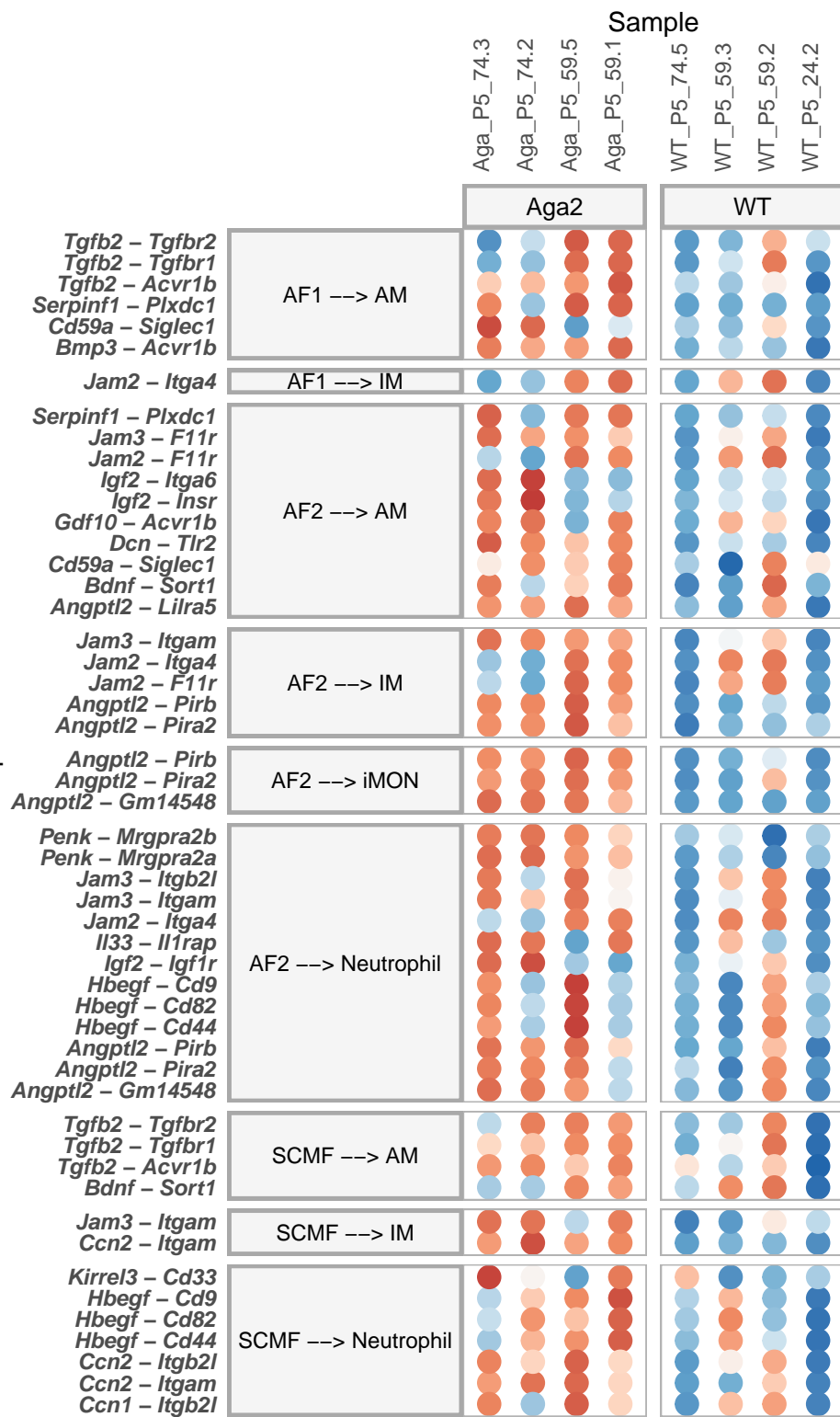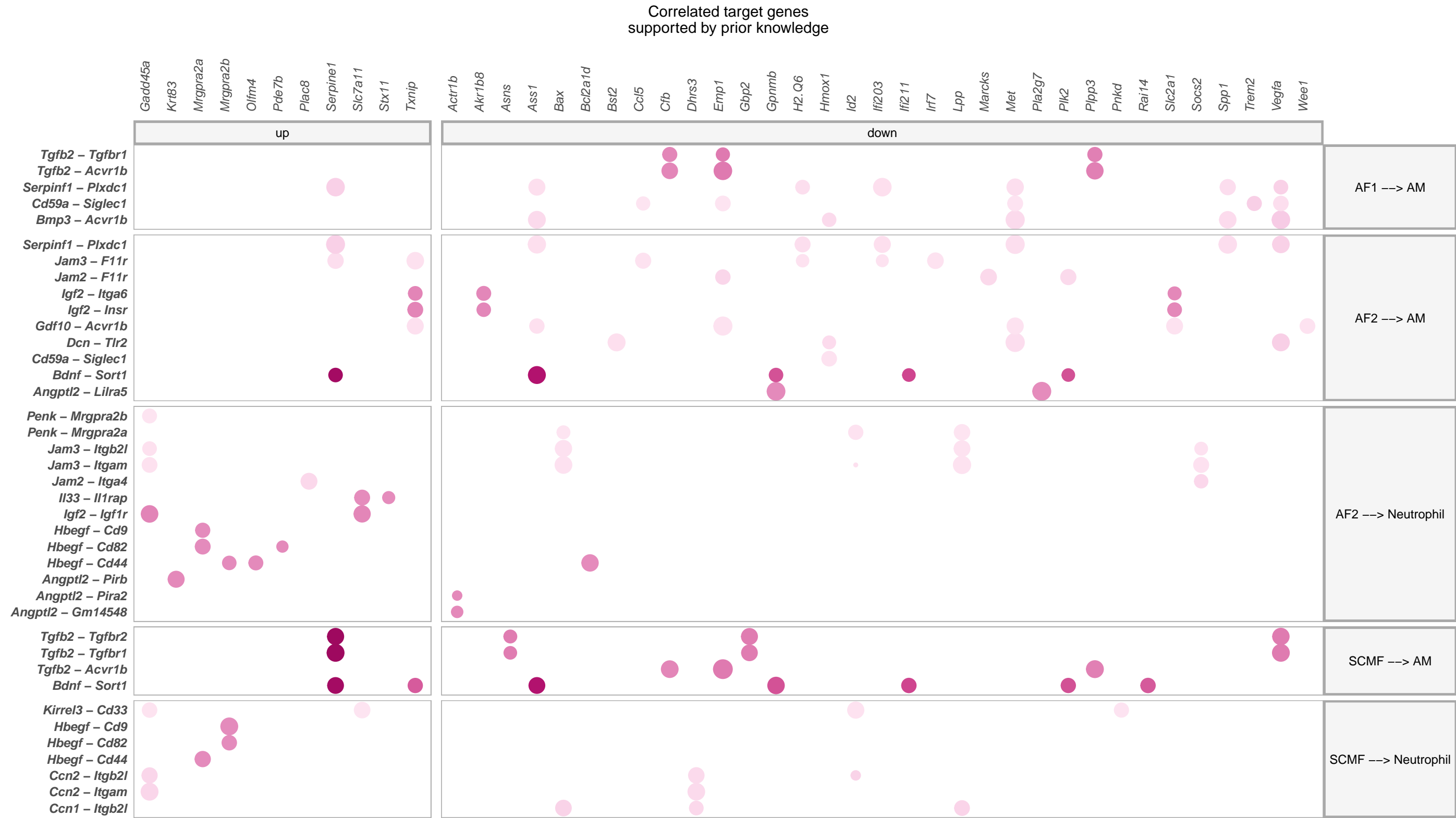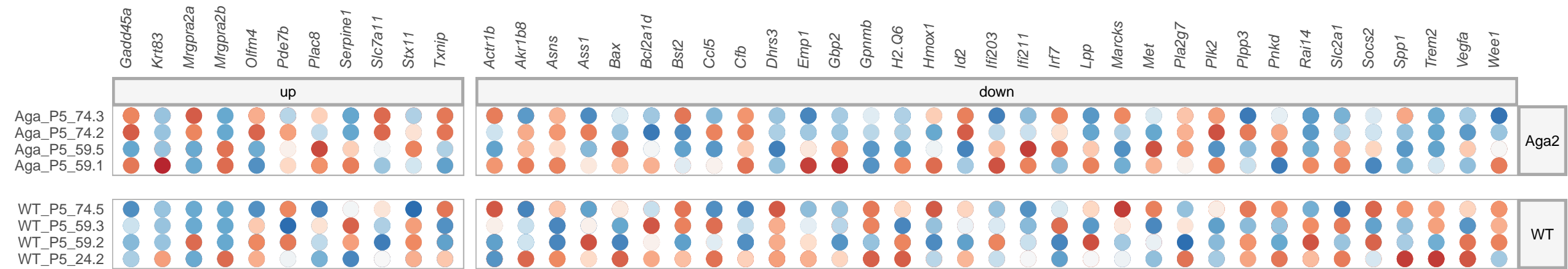

Supplement: Supplementary file 1 [file DataSheet7.pdf]
