# Supplementary material for: Lung scRNA-seq reveals chronic inflammation and emphysemous phenotype in mice with osteogenesis imperfecta
Source: Front Genet. 2026 Feb 26;17:1713393. doi: 10.3389/fgene.2026.1713393 (PMC12978693; doi:10.3389/fgene.2026.1713393)
Supplement: Supplementary file 2 [file DataSheet9.pdf]

Supplemental methods:

Generation of Mice

Aga2+/– animals were received as a gift from the Jacobsen laboratory at Harvard Medical School in Boston, Massachusetts and maintained on a C57BL/6J background (18). The *Aga2+/–* colony was maintained through mutant alleles only passed through males. UCLA follows the PHS Policy that requires that all institutions base their animal care and use programs on the Guide for the Care and Use of Laboratory Animals, and that euthanasia be consistent with the American Veterinary Medical Association (AVMA) Guidelines on Euthanasia. The animals were monitored at least once daily and once during the weekend by personnel associated with this proposal, and by the veterinary and technical staff of the UCLA Department of Laboratory Animal Medicine. Fresh water and food were supplied for consumption as needed, and visibly unhealthy animals were removed immediately and euthanized by inhalation. Sentinel animals are used for detailed serological testing to ensure that the colony remains pathogen free.

Tissue collection, dissociation for single cell RNAseq

Lung tissue was perfused with sterile saline from the right to the left ventricle of the heart and subsequently inflated via a catheter in the trachea by an enzyme mix containing Collagenase/Dispase (2.5 mg/ml), and DNase (30 µg/ml) in PBS. After tying off the trachea, the lung was removed and immediately minced to small pieces (approximately 1 mm^2). The tissue was transferred into 4 ml enzyme mix for enzymatic digestion for 30-60 min at 37 °C. Enzyme activity was inhibited by adding 5 ml of phosphate-buffered saline (PBS) supplemented with 10% fetal calf serum (FCS). Dissociated cells in suspension were passed through a 70 µm

strainer and centrifuged at 500 × g for 5 min at 4 °C. Red blood cell lysis (Thermo Fisher 00-4333-57) was done for 2 min and stopped with 10% FCS in PBS. After another centrifugation for 5 min at 500 × g (4 °C) the cells were counted and critically assessed for single-cell separation and viability. Cell numbers and viability were quantified and only samples with great than 80% cell viability were used for subsequent cell encapsulation and library construction. Cells were encapsulated into emulsion droplets by Chromium Controller (10x Genomics) and libraries were constructed using the 10X Genomics Chromium Single Cell 3′ v3 reagent kit. cDNA libraries were sequenced in a Novaseq S2 sequencer using using 100-cycle paired-end reads, generating ~45,000 reads per cell with a total of ~10,000 cells per sample.

Supplemental Figures

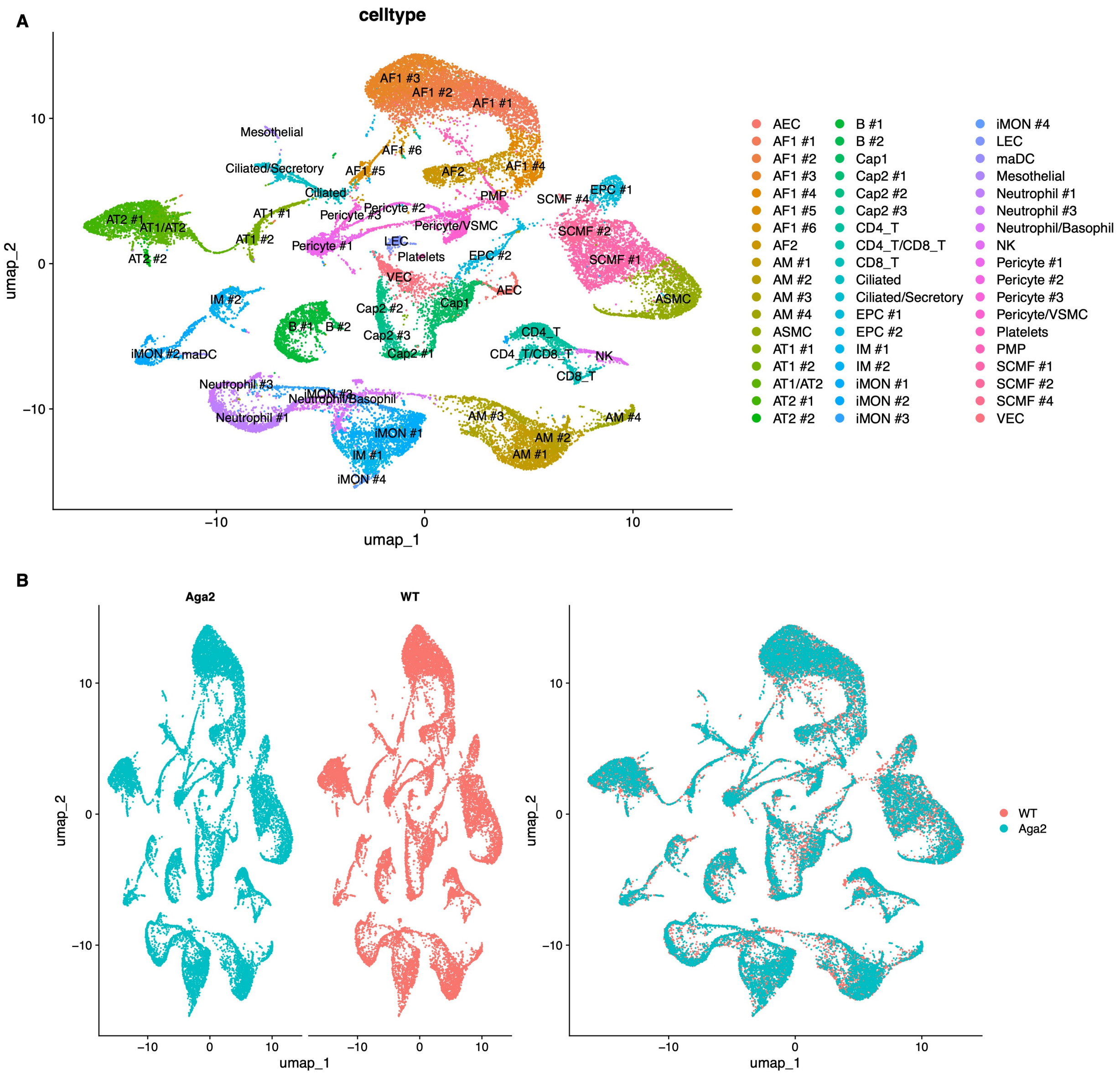

Supplemental Figure 1: scRNA-seq analysis of P5 WT and Aga2 lungs (A,B) Cell clusters from scRNA-Seq analysis visualized by uniform manifold approximation and projection (UMAP). N = 4 for each genotype (B) UMAP plots of Aga2 and WT samples split (left) and overlayed (right) indicating a roughly equal distribution of both genotypes in all clusters.

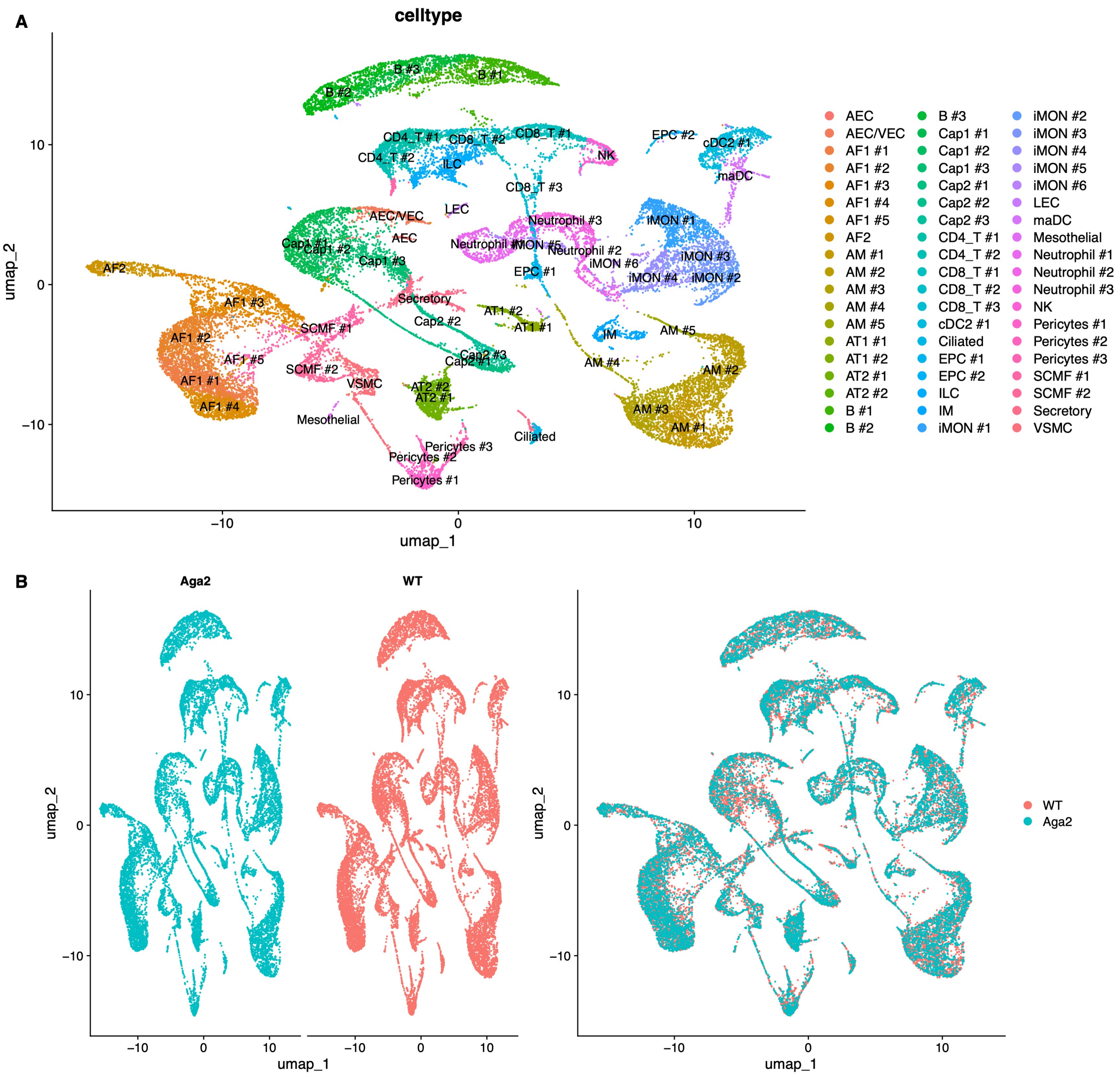

Supplemental Figure 2: scRNA-seq analysis of P28 WT and Aga2 lungs (A,B) Cell clusters from scRNA-Seq analysis visualized by uniform manifold approximation and projection (UMAP). N = 4 for each genotype (B) UMAP plots of Aga2 and WT samples split (left) and overlayed (right) indicating a roughly equal distribution of both genotypes in all clusters.

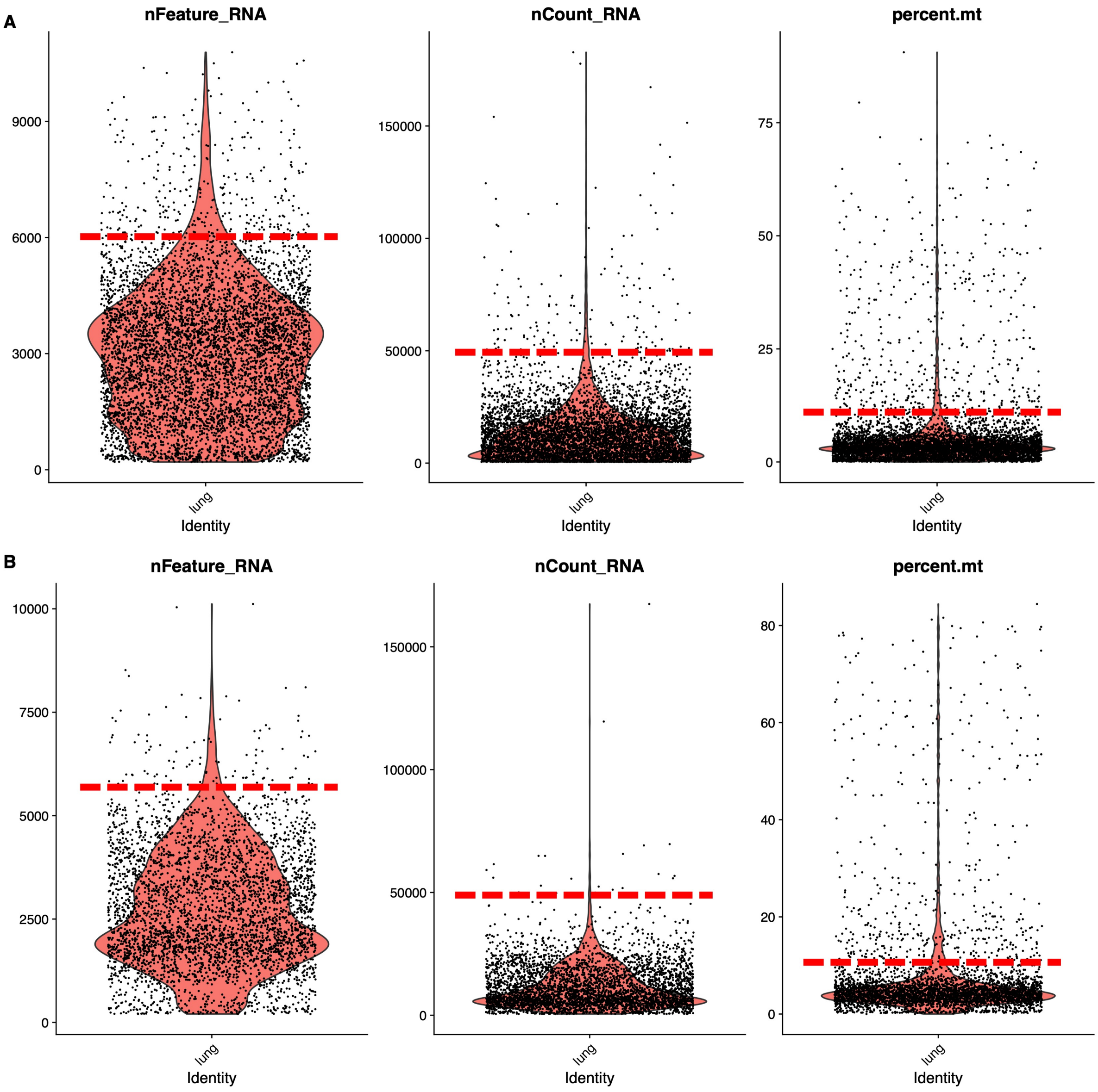

Supplemental Figure 3: Violin plots of (A) p5 and (B) p28 gene counts per cell (nFeature_RNA), RNA counts per cell (nCount_RNA), and percentage of mitochondrial genes per cell (percent.mt). Red dashed lines indicate the upper threshold used for initial QC of scRNAseq data prior to analysis.

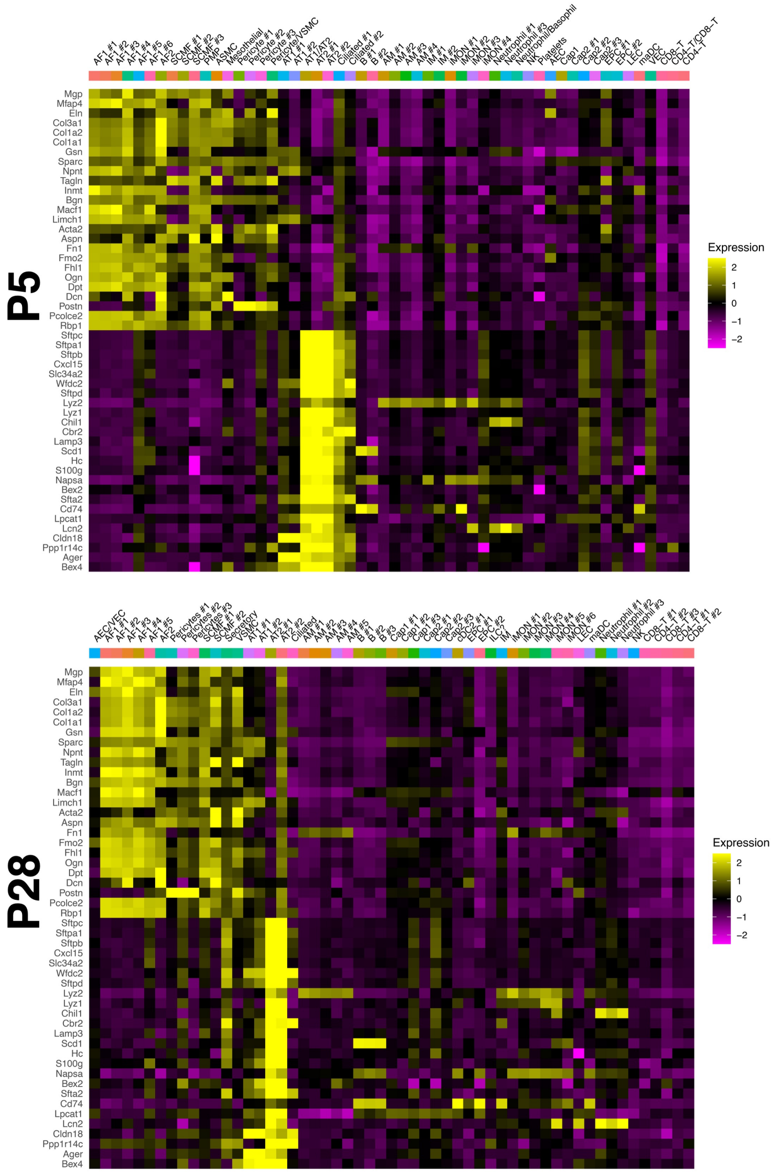

Supplemental Figure 4: Expression heatmaps from p5 and p28 scRNAseq of WT and Aga2 lung issues showing top differentially expressed genes in each cluster.

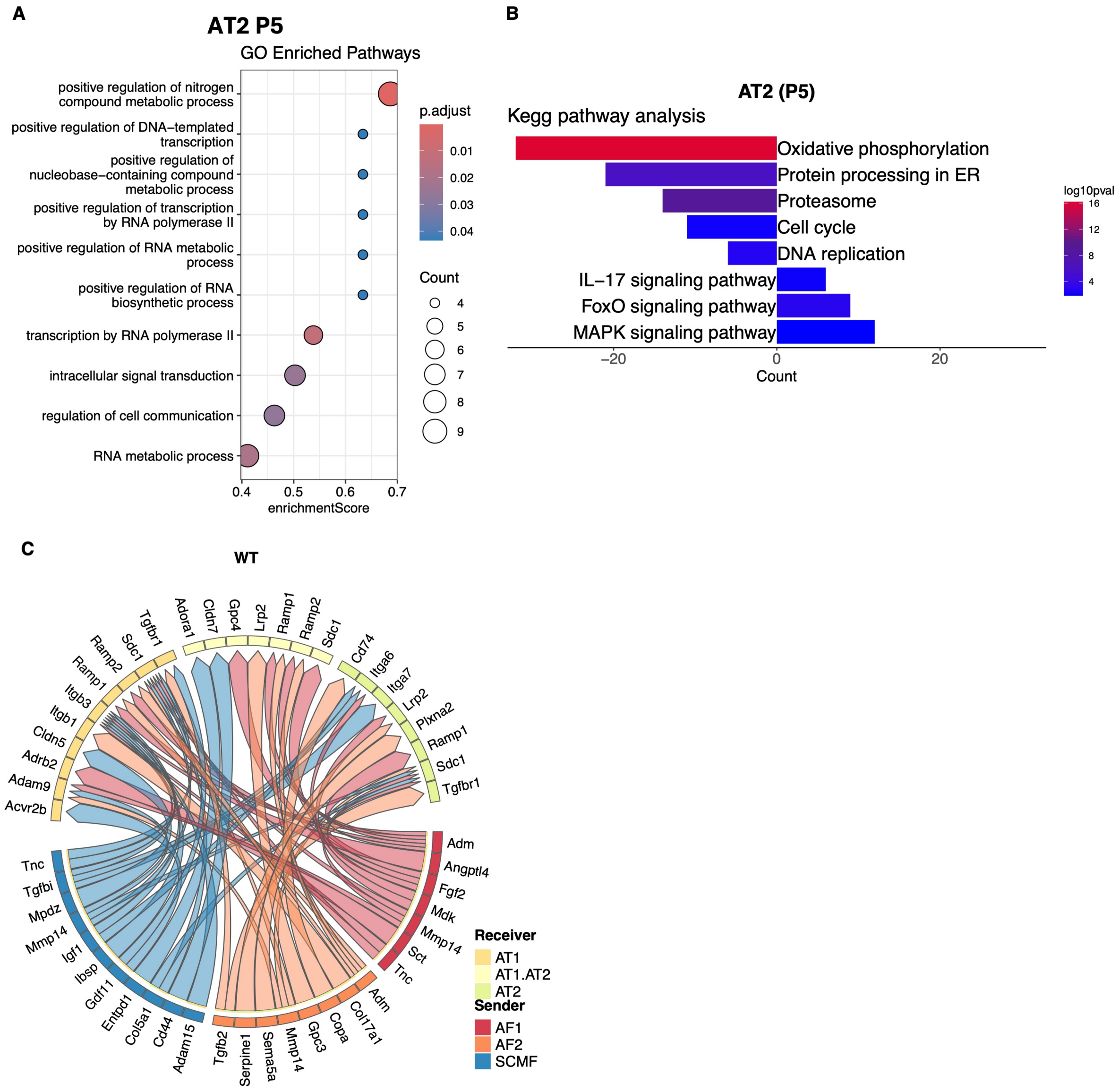

Supplemental Figure 5: (A,B) Top relevant enriched GO and KEGG pathway analysis terms in differential expression between WT and Aga2 in p5 AT2 epithelial cells. (C) Circos plot showing inferred downregulated in Aga2 cell communication via MultiNicheNet analysis. Receiver epithelial cells with decreased expression of signaling receptors and downstream targets are connected to the fibroblast sender cell types expressing ligands predicted to promote this response. Ligands expressed by the same cell population are colored the same.

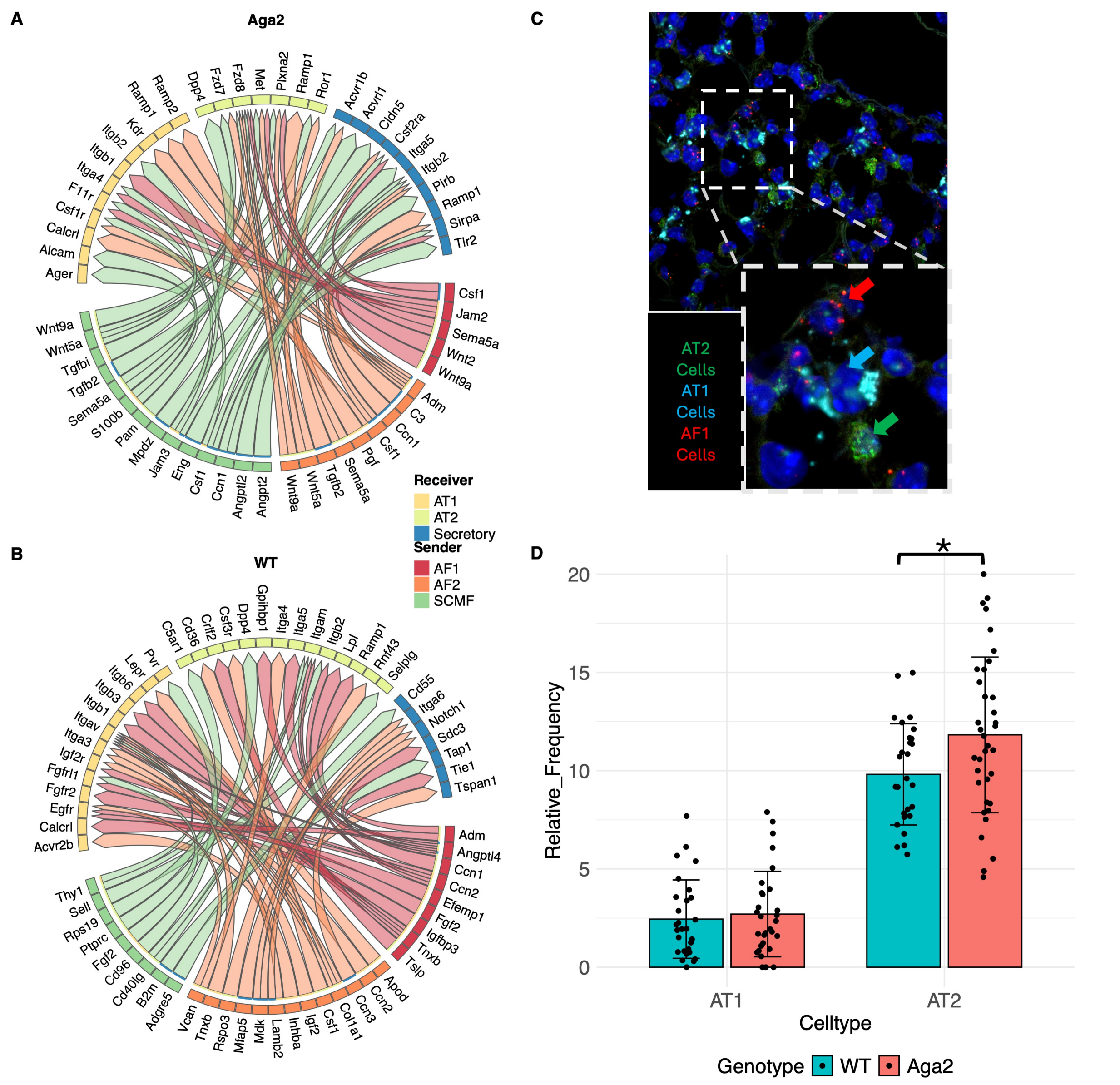

Supplemental figure 6: (A,B) Circos plot showing inferred upregulated (A) and downregulated (B) pathways in Aga2 cell communication via MultiNicheNet analysis. Receiver epithelial cells with decreased expression of signaling receptors and downstream targets are connected to the fibroblast sender cell types expressing ligands predicted to promote this response. Ligands expressed by the same cell population are colored the same. (C) RNAscope for Sftpc (green, AT2) costained with Ager (cyan, AT1), Tcf21 (red, AF1), and DAPI (blue) indicating the close physical proximity of fibroblasts and epithelial cells in the P28 mouse lung. (D) Quantification of AT1 and AT2 cell numbers at P28 in WT and Aga2 lungs. * = p<0.05, bars represent mean +/- SD. N = 3 WT, 4 Aga2 with 10 fields quantified in each biological replicate.

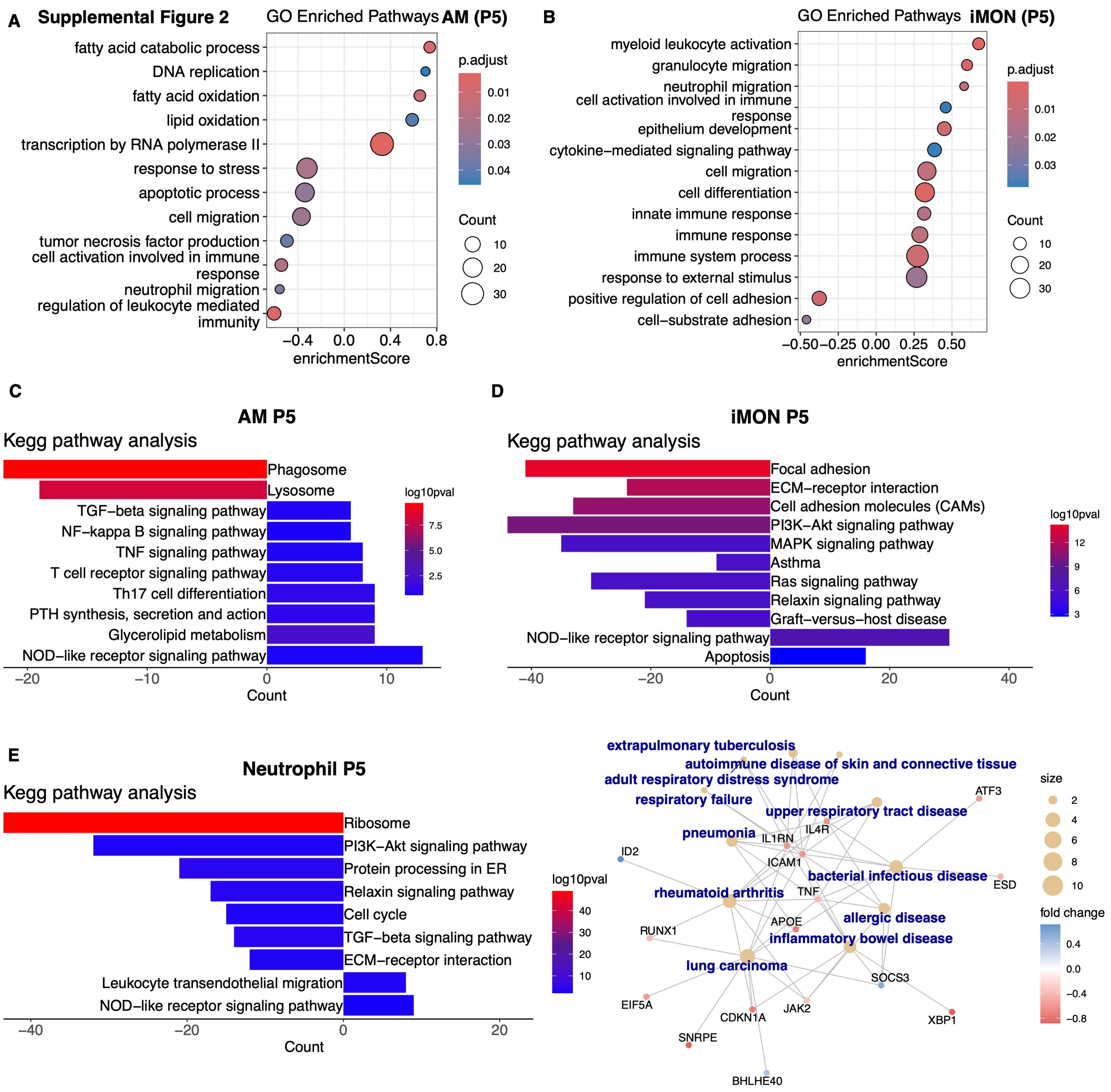

Supplemental Figure 7: (A-E) Top relevant enriched GO and KEGG pathway analysis terms in differential expression between WT and Aga2 in p5 immune cells. (F) CNET plot of top relevant enriched human diseases in differential expression between WT and Aga2 in p5 neutrophils.

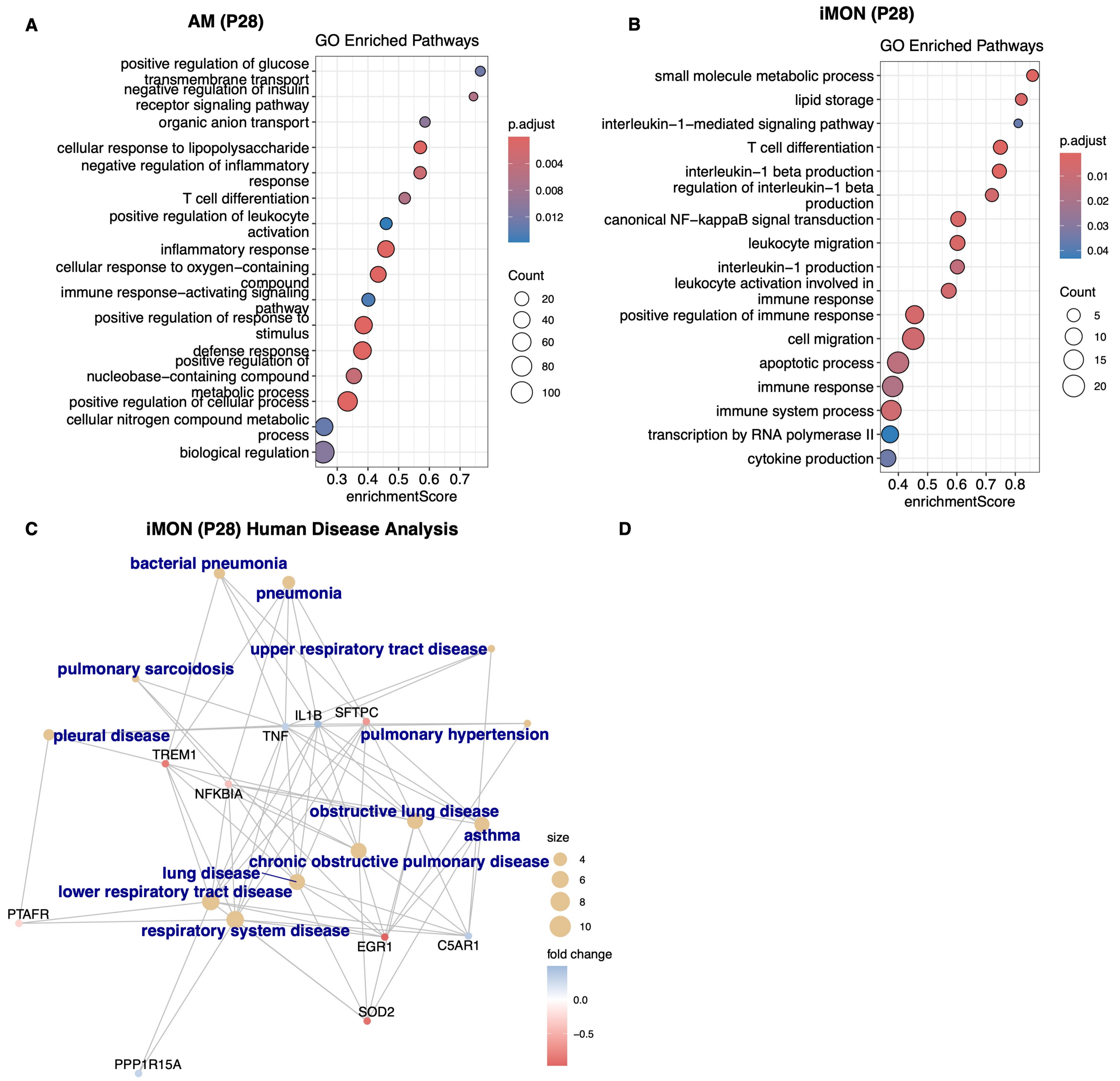

Supplemental Figure 8: (A,B) Top relevant enriched GO pathway analysis terms in differential expression between WT and Aga2 in p28 immune cells. (C) CNET plot of top relevant enriched human diseases in differential expression between WT and Aga2 in p28 iMONs.

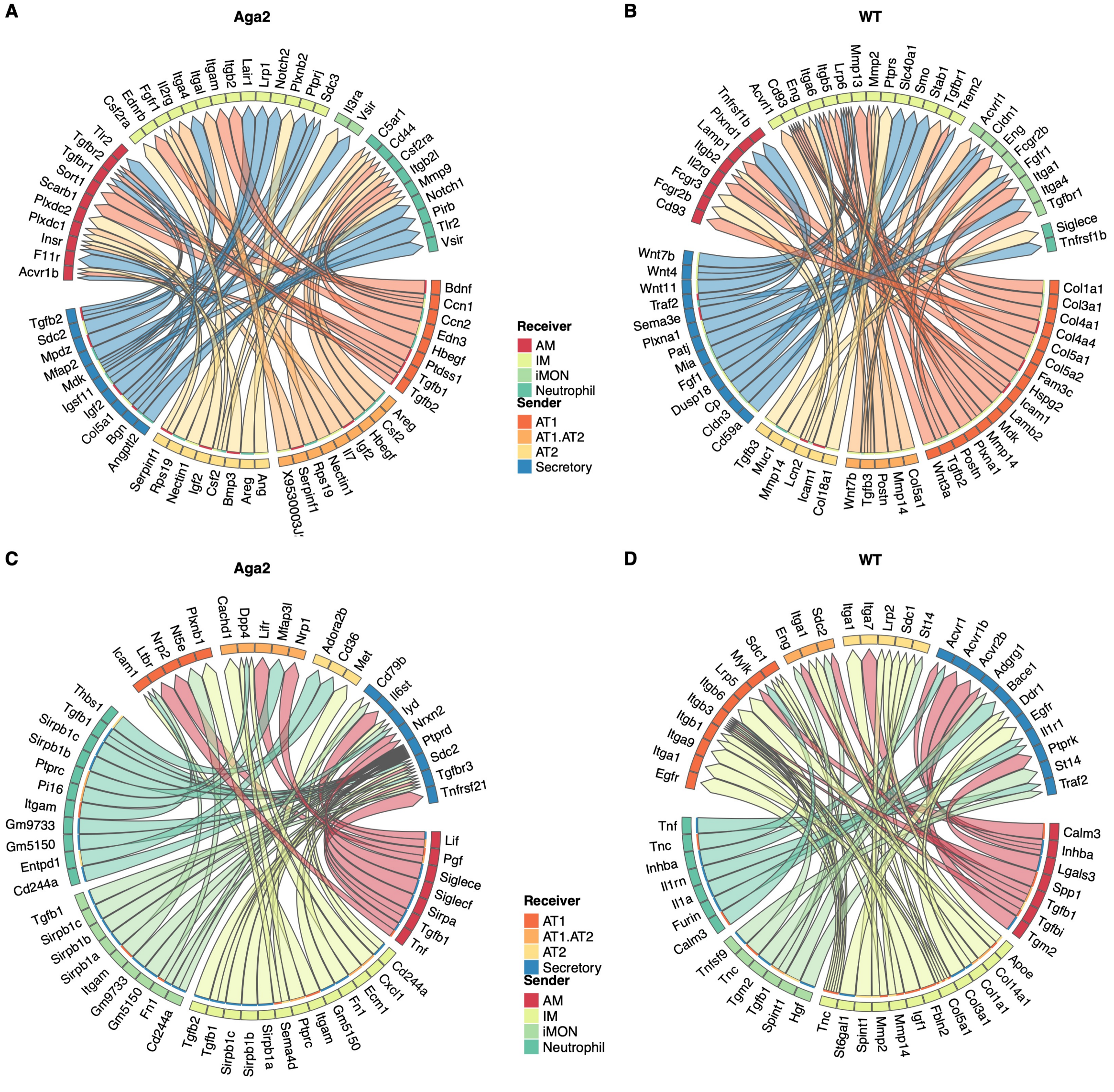

Supplemental Figure 9: (A,B) Circos plot showing inferred upregulated (A) and downregulated (B) in Aga2 p5 cell communication via MultiNicheNet analysis. Receiver immune cells with differential expression of signaling receptors and downstream targets are connected to the epithelial sender cell types expressing ligands predicted to promote this response. Ligands expressed by the same cell population are colored the same. (C,D) Circos plot showing inferred upregulated (C) and downregulated (D) in Aga2 p5 cell communication via MultiNicheNet analysis. Receiver epithelial cells with differential expression of signaling receptors and downstream targets are connected to the immune sender cell types expressing ligands predicted to promote this response. Ligands expressed by the same cell population are colored the same.

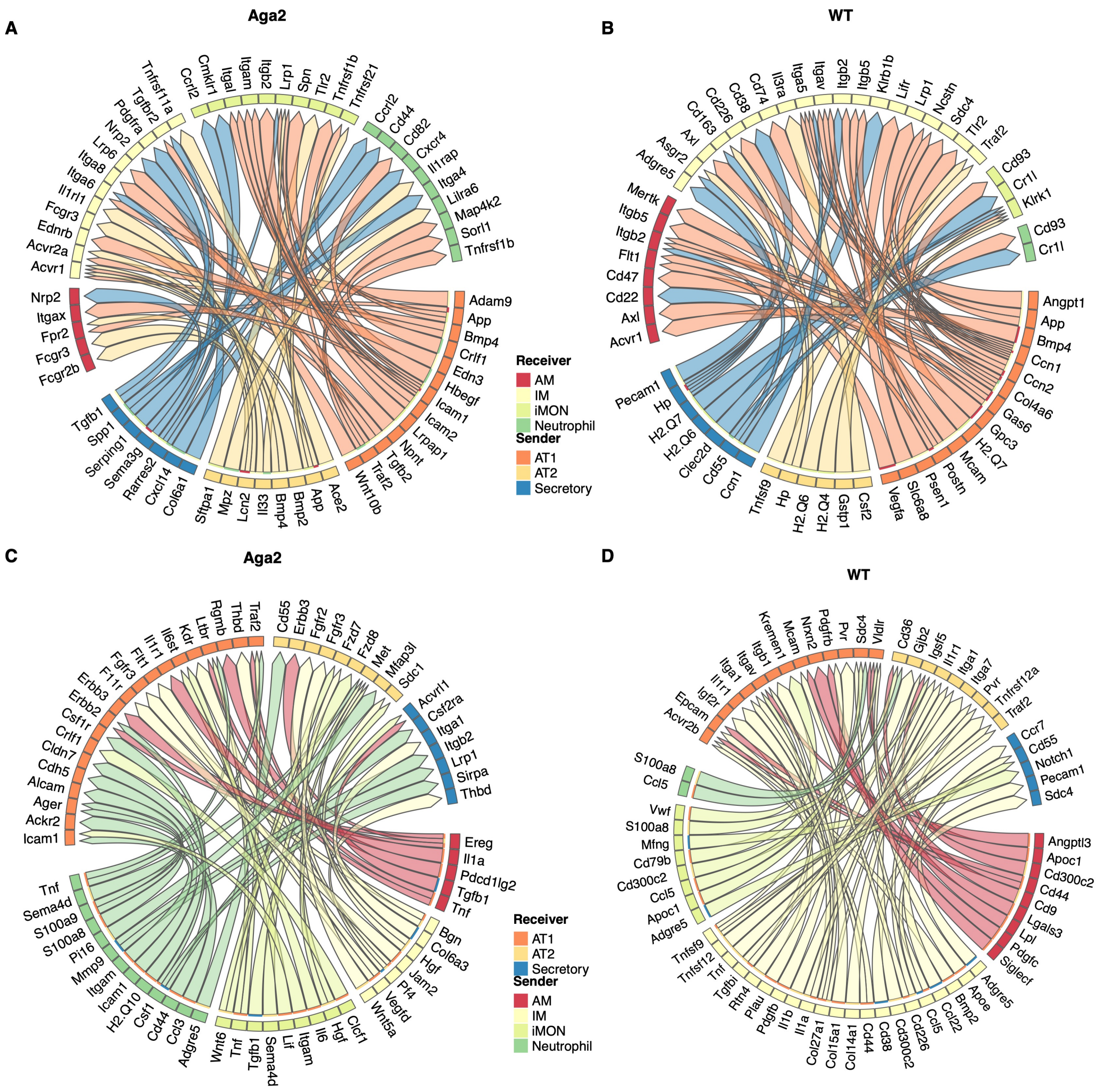

Supplemental Figure 10: (A,B) Circos plot showing inferred upregulated (A) and downregulated (B) in Aga2 p28 cell communication via MultiNicheNet analysis. Receiver immune cells with differential expression of signaling receptors and downstream targets are connected to the epithelial sender cell types expressing ligands predicted to promote this response. Ligands expressed by the same cell population are colored the same. (C,D) Circos plot showing inferred upregulated (C) and downregulated (D) in Aga2 p28 cell communication via MultiNicheNet analysis. Receiver epithelial cells with differential expression of signaling receptors and downstream targets are connected to the immune sender cell types expressing ligands predicted to promote this response. Ligands expressed by the same cell population are colored the same.

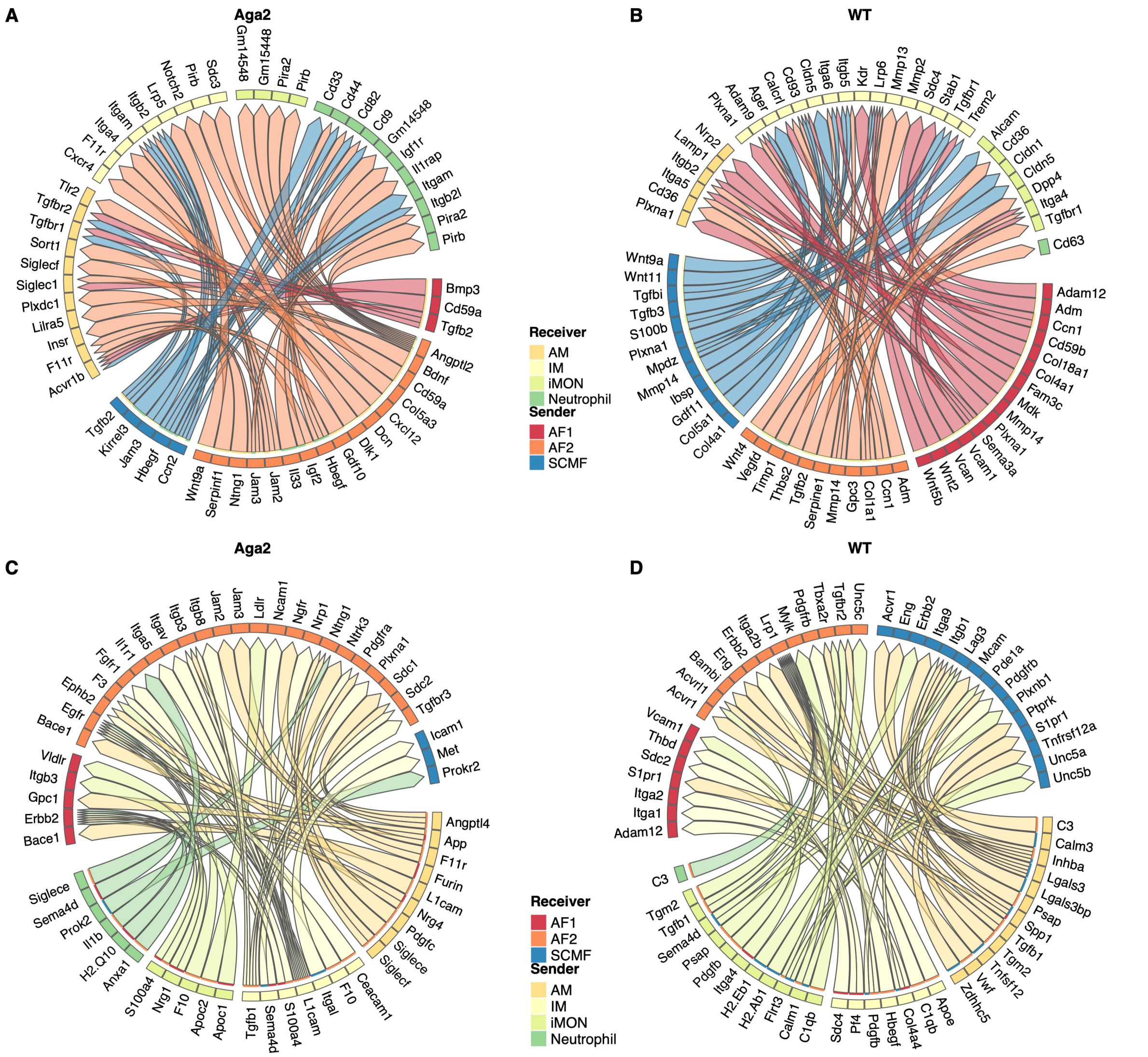

Supplemental Figure 11: (A,B) Circos plot showing inferred upregulated (A) and downregulated (B) in Aga2 p5 cell communication via MultiNicheNet analysis. Receiver immune cells with differential expression of signaling receptors and downstream targets are connected to the fibroblast sender cell types expressing ligands predicted to promote this response. Ligands expressed by the same cell population are colored the same. (C,D) Circos plot showing inferred upregulated (C) and downregulated (D) in Aga2 p5 cell communication via MultiNicheNet analysis. Receiver fibroblast cells with differential expression of signaling receptors and downstream targets are connected to the immune sender cell types expressing ligands predicted to promote this response. Ligands expressed by the same cell population are colored the same.

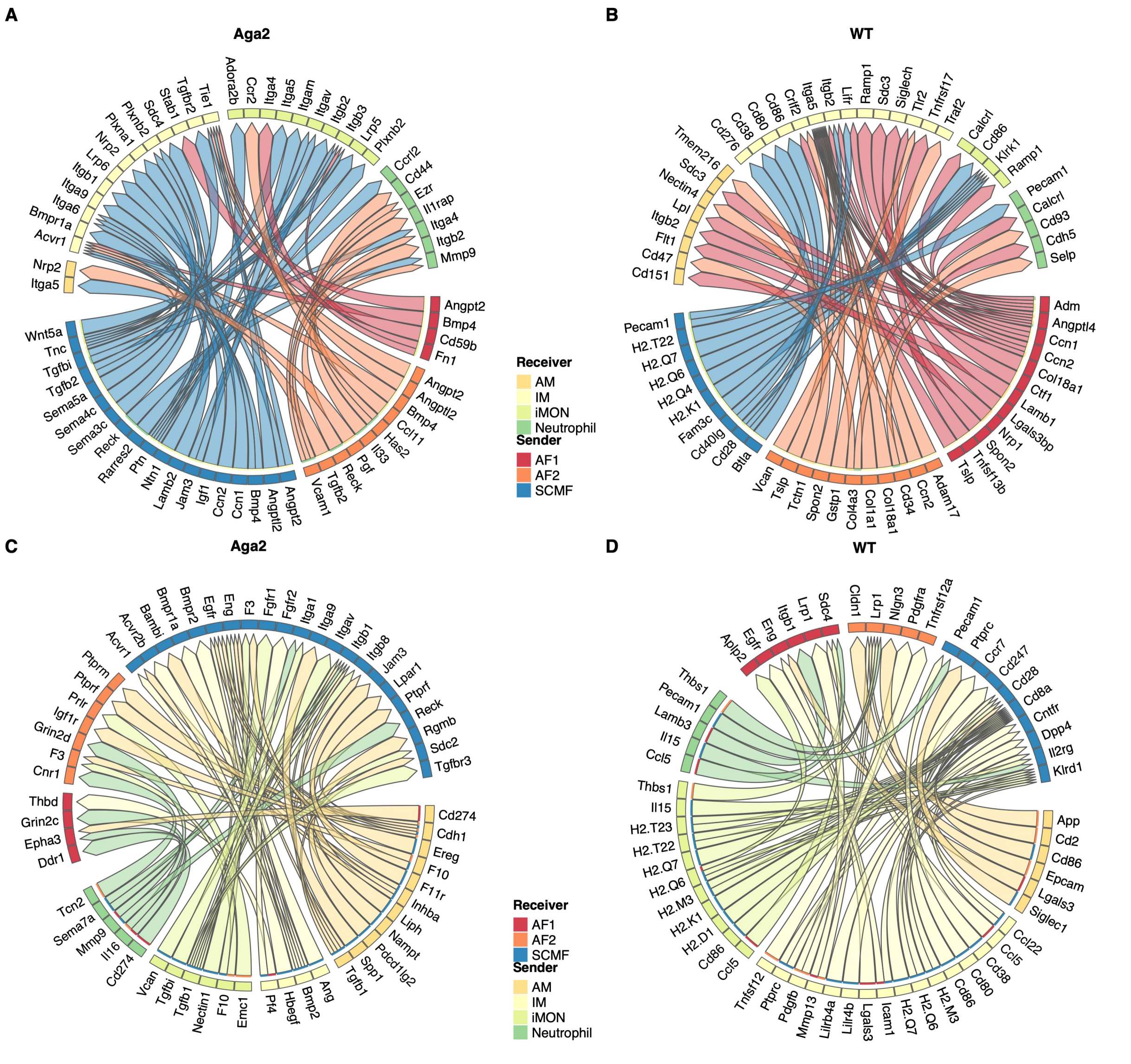

Supplemental Figure 12: (A,B) Circos plot showing inferred upregulated (A) and downregulated (B) in Aga2 p28 cell communication via MultiNicheNet analysis. Receiver immune cells with differential expression of signaling receptors and downstream targets are connected to the fibroblast sender cell types expressing ligands predicted to promote this response. Ligands expressed by the same cell population are colored the same. (C,D) Circos plot showing inferred upregulated (C) and downregulated (D) in Aga2 p28 cell communication via MultiNicheNet analysis. Receiver fibroblast cells with differential expression of signaling receptors and downstream targets are connected to the immune sender cell types expressing ligands predicted to promote this response. Ligands expressed by the same cell population are colored the same.

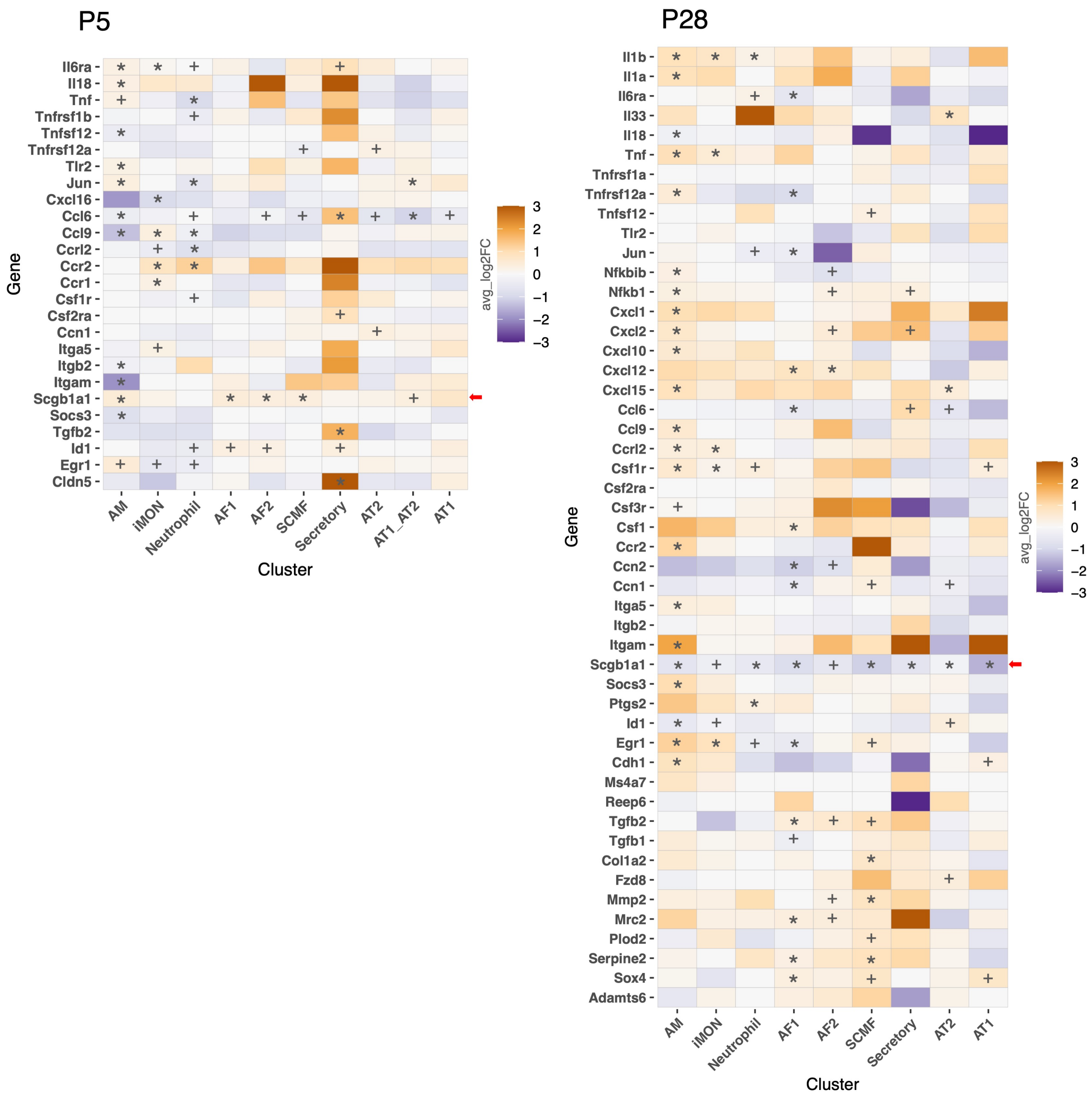

Supplemental Figure 13: p5 and p28 heatmaps showing differential expression of inflammation markers in p5 and p28 lung immune, fibroblast, and epithelial cells. * = adjusted Pvalue<0.05, + = non-adjust Pvalue<0.05.

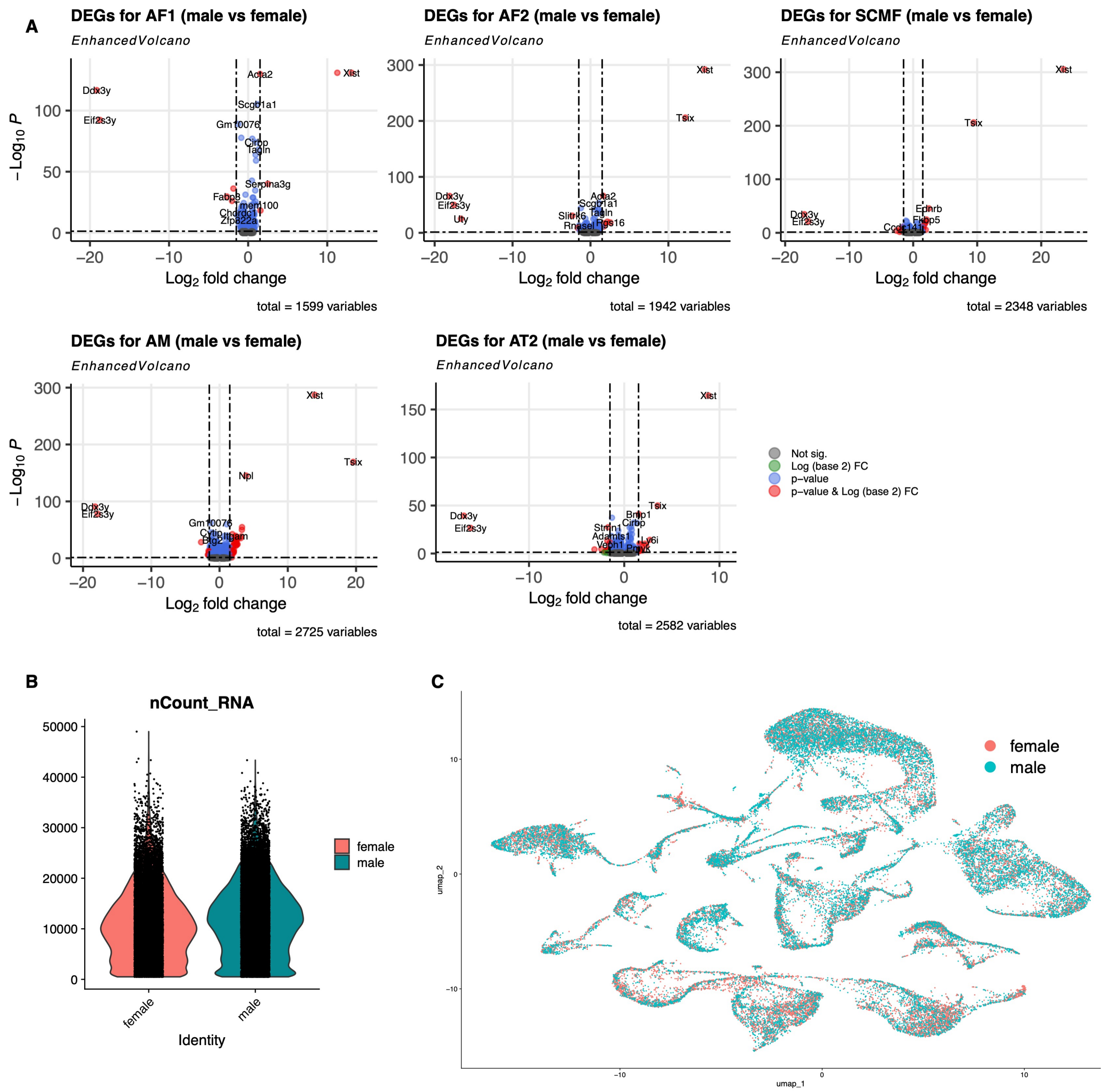

Supplemental Figure 14: Analysis of sex differences in p5 lungs. (A) Volcano plots of differentially expressed genes (DEGs) in a male vs female comparison in a select number of clusters. In each plot, genes on the left are enriched in male samples and genes on the right are enriched in female samples. An adjusted p-value of less than 0.05 and a Log (base 2) fold change of greater than 1 was considered significantly different. (B) Comparison of RNA counts per cell in males vs females indicating no significant difference. (C) UMAP projection of p5 scRNAseq data showing cells belonging to males or females evenly distributed in all clusters.

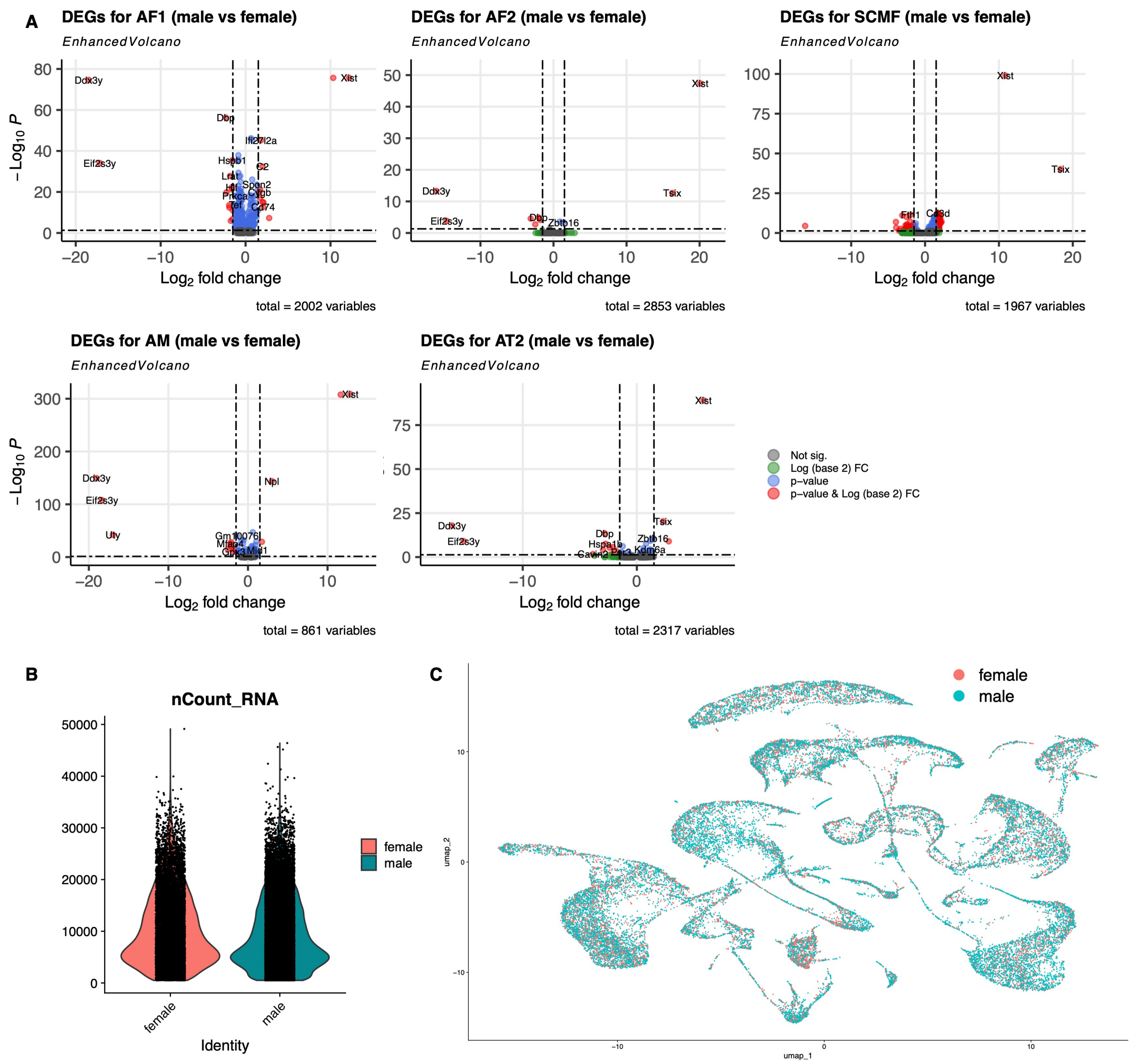

Supplemental Figure 15: Analysis of sex differences in p28 lungs. (A) Volcano plots of differentially expressed genes (DEGs) in a male vs female comparison in a select number of clusters. In each plot, genes on the left are enriched in male samples and genes on the right are enriched in female samples. An adjusted p-value of less than 0.05 and a Log (base 2) fold change of greater than 1 was considered significantly different. (B) Comparison of RNA counts per cell in males vs females indicating no significant difference. (C) UMAP projection of p28 scRNAseq data showing cells belonging to males or females evenly distributed in all
