# Supplementary material for: Lung scRNA-seq reveals chronic inflammation and emphysemous phenotype in mice with osteogenesis imperfecta
Source: Front Genet. 2026 Feb 26;17:1713393. doi: 10.3389/fgene.2026.1713393 (PMC12978693; doi:10.3389/fgene.2026.1713393)
Supplement: Supplementary file 6 [file DataSheet8.pdf]

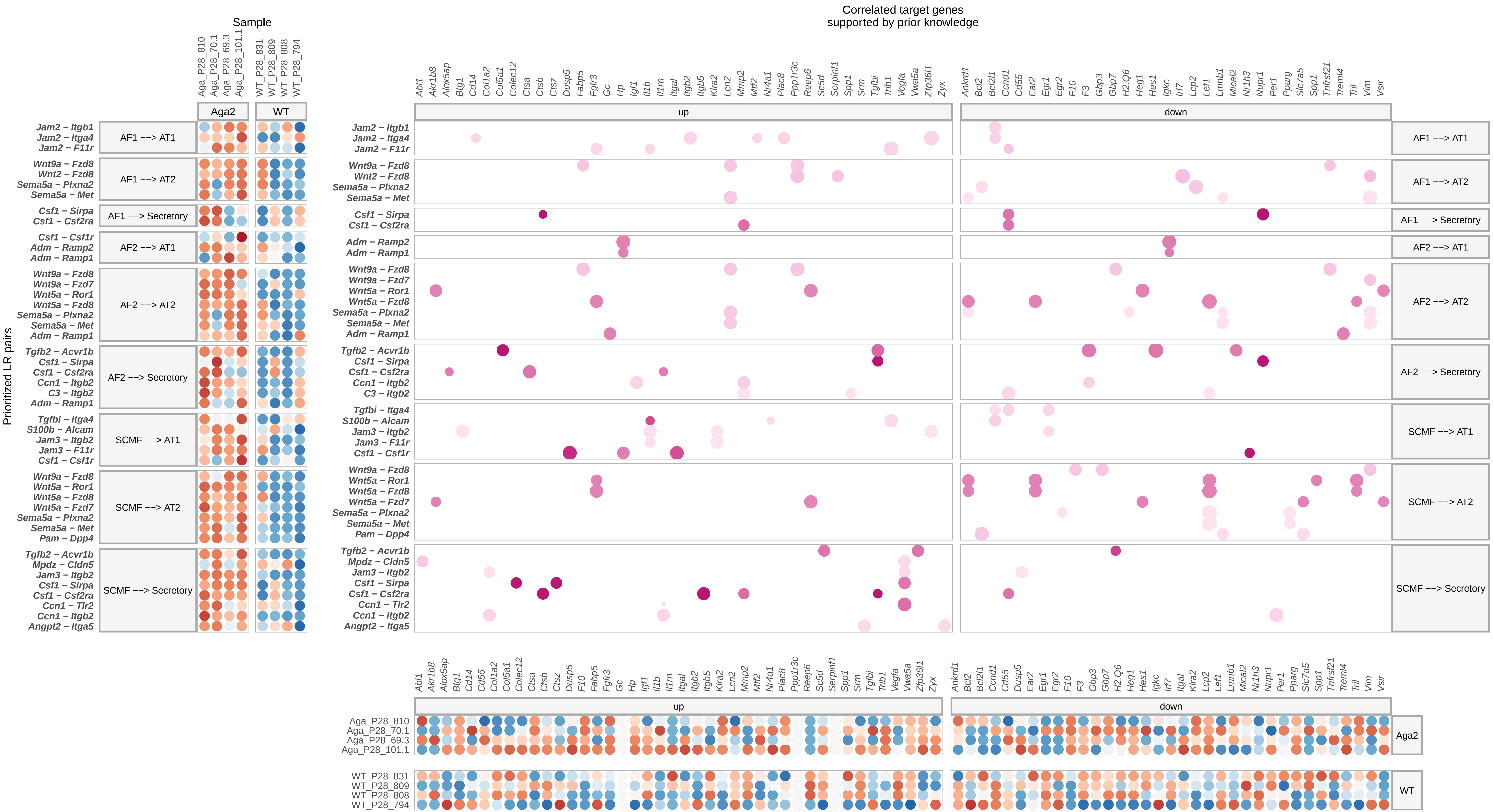

Sample
Correlated target genes
supported by prior knowledge
Aga_P28_810
Aga_P28_70.1
Aga_P28_69.3
Aga_P28_101.1
WT_P28_831
WT_P28_809
WT_P28_808
WT_P28_794
Aga2
WT
up
down
Abl1 Akr1b8 Alox5ap Btg1 Cd14 Col1a2 Col5a1 Colec12 Ctsa Ctsb Ctsz Dusp5 Fabp5 Fgfr3 Gc Hp Igf1 Il1b Il1rn Itgal Itgb2 Itgb5 Klra2 Lcn2 Mmp2 Mtf2 Nr4a1 Plac8 Ppp1r3c Reep6 Sc5d Serpinf1 Spp1 Srm Tgfbi Trib1 Vegfa Vwa5a Zfp36l1 Zyx
Ankrd1 Bcl2 Bcl2l1 Ccnd1 Cd55 Ear2 Egr1 Egr2 F10 F3 Gbp3 Gbp7 H2.Q6 Heg1 Hes1 Igkc Irf7 Lcp2 Lef1 Lmnb1 Mical2 Nr1h3 Nupr1 Per1 Pparg Slc7a5 Spp1 Tnfrsf21 Treml4 Tril Vim Vsir
Prioritized LR pairs
AF1 --> AT1
Jam2 – Itgb1
Jam2 – Itga4
Jam2 – F11r
AF1 --> AT2
Wnt9a – Fzd8
Wnt2 – Fzd8
Sema5a – Plxna2
Sema5a – Met
AF1 --> Secretory
Csf1 – Sirpa
Csf1 – Csf2ra
AF2 --> AT1
Csf1 – Csf1r
Adm – Ramp2
Adm – Ramp1
AF2 --> AT2
Wnt9a – Fzd8
Wnt9a – Fzd7
Wnt5a – Ror1
Wnt5a – Fzd8
Sema5a – Plxna2
Sema5a – Met
Adm – Ramp1
AF2 --> Secretory
Tgfb2 – Acvr1b
Csf1 – Sirpa
Csf1 – Csf2ra
Ccn1 – Itgb2
C3 – Itgb2
Adm – Ramp1
SCMF --> AT1
Tgfbi – Itga4
S100b – Alcam
Jam3 – Itgb2
Jam3 – F11r
Csf1 – Csf1r
SCMF --> AT2
Wnt9a – Fzd8
Wnt5a – Ror1
Wnt5a – Fzd8
Wnt5a – Fzd7
Sema5a – Plxna2
Sema5a – Met
Pam – Dpp4
SCMF --> Secretory
Tgfb2 – Acvr1b
Mpdz – Cldn5
Jam3 – Itgb2
Csf1 – Sirpa
Csf1 – Csf2ra
Ccn1 – Tlr2
Ccn1 – Itgb2
Angpt2 – Itga5
Abl1 Akr1b8 Alox5ap Btg1 Cd14 Cd55 Col1a2 Col5a1 Colec12 Ctsa Ctsb Ctsz Dusp5 F10 Fabp5 Fgfr3 Gc Hp Igf1 Il1b Il1rn Itgal Itgb2 Itgb5 Klra2 Lcn2 Mmp2 Mtf2 Nr4a1 Plac8 Ppp1r3c Reep6 Sc5d Serpinf1 Spp1 Srm Tgfbi Trib1 Vegfa Vwa5a Zfp36l1 Zyx
Ankrd1 Bcl2 Bcl2l1 Ccnd1 Cd55 Dusp5 Ear2 Egr1 Egr2 F10 F3 Gbp3 Gbp7 H2.Q6 Heg1 Hes1 Igkc Irf7 Itgal Klra2 Lcp2 Lef1 Lmnb1 Mical2 Nr1h3 Nupr1 Per1 Pparg Slc7a5 Spp1 Tnfrsf21 Treml4 Tril Vim Vsir

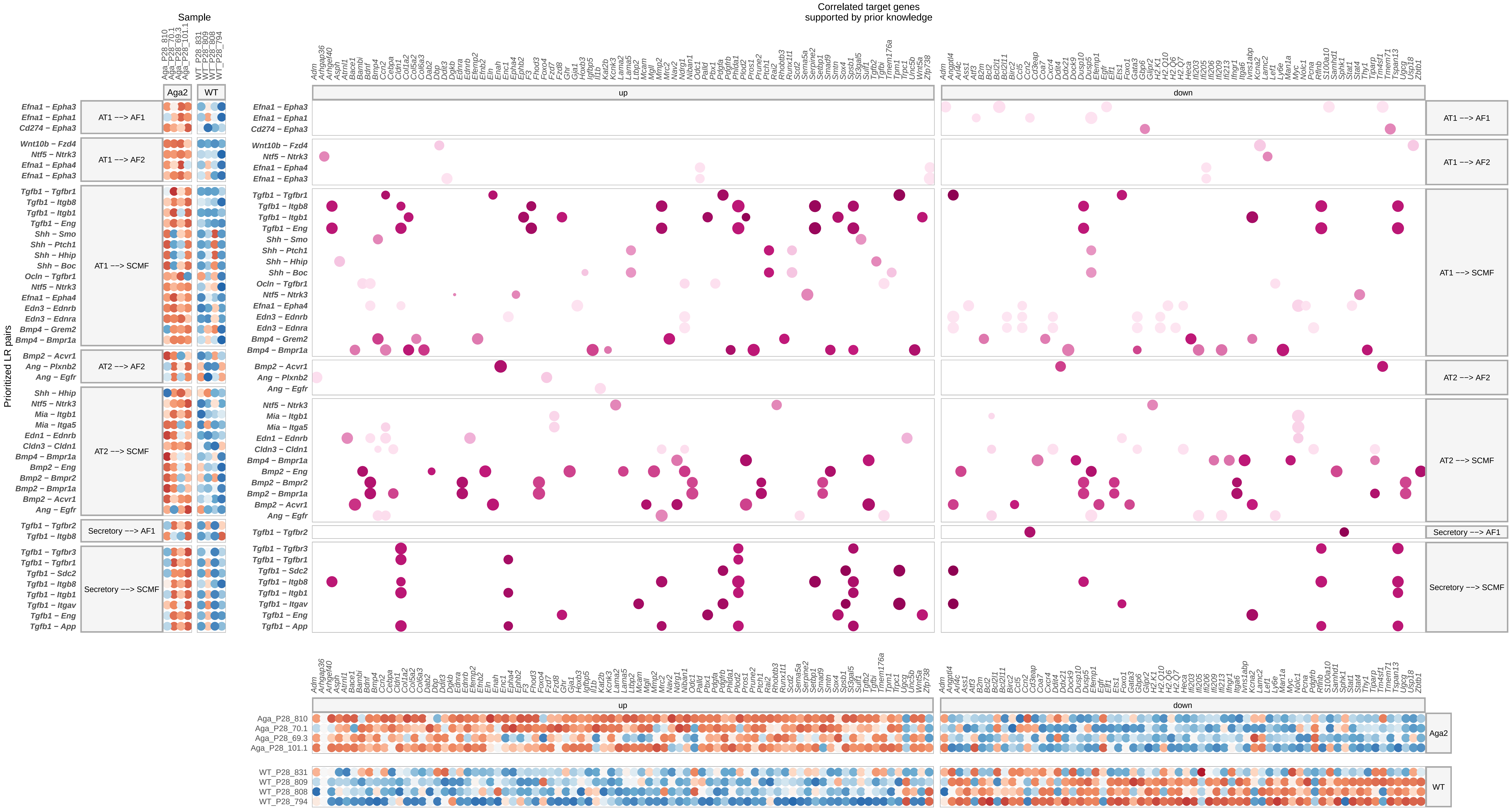
Sample
Aga_P28_810
Aga_P28_70.1
Aga_P28_69.3
Aga_P28_101.1
WT_P28_831
WT_P28_809
WT_P28_808
WT_P28_794
Aga2
WT
Correlated target genes
supported by prior knowledge
up
down
Prioritized LR pairs
AT1 --> AF1
Efna1 – Epha3
Efna1 – Epha1
Cd274 – Epha3
AT1 --> AF2
Wnt10b – Fzd4
Ntf5 – Ntrk3
Efna1 – Epha4
Efna1 – Epha3
AT1 --> SCMF
Tgfb1 – Tgfbr1
Tgfb1 – Itgb8
Tgfb1 – Itgb1
Tgfb1 – Eng
Shh – Smo
Shh – Ptch1
Shh – Hhip
Shh – Boc
Ocln – Tgfbr1
Ntf5 – Ntrk3
Efna1 – Epha4
Edn3 – Ednrb
Edn3 – Ednra
Bmp4 – Grem2
Bmp4 – Bmpr1a
AT2 --> AF2
Bmp2 – Acvr1
Ang – Plxnb2
Ang – Egfr
AT2 --> SCMF
Shh – Hhip
Ntf5 – Ntrk3
Mia – Itgb1
Mia – Itga5
Edn1 – Ednrb
Cldn3 – Cldn1
Bmp4 – Bmpr1a
Bmp2 – Eng
Bmp2 – Bmpr2
Bmp2 – Bmpr1a
Bmp2 – Acvr1
Ang – Egfr
Secretory --> AF1
Tgfb1 – Tgfbr2
Tgfb1 – Itgb8
Secretory --> SCMF
Tgfb1 – Tgfbr3
Tgfb1 – Tgfbr1
Tgfb1 – Sdc2
Tgfb1 – Itgb8
Tgfb1 – Itgb1
Tgfb1 – Itgav
Tgfb1 – Eng
Tgfb1 – App

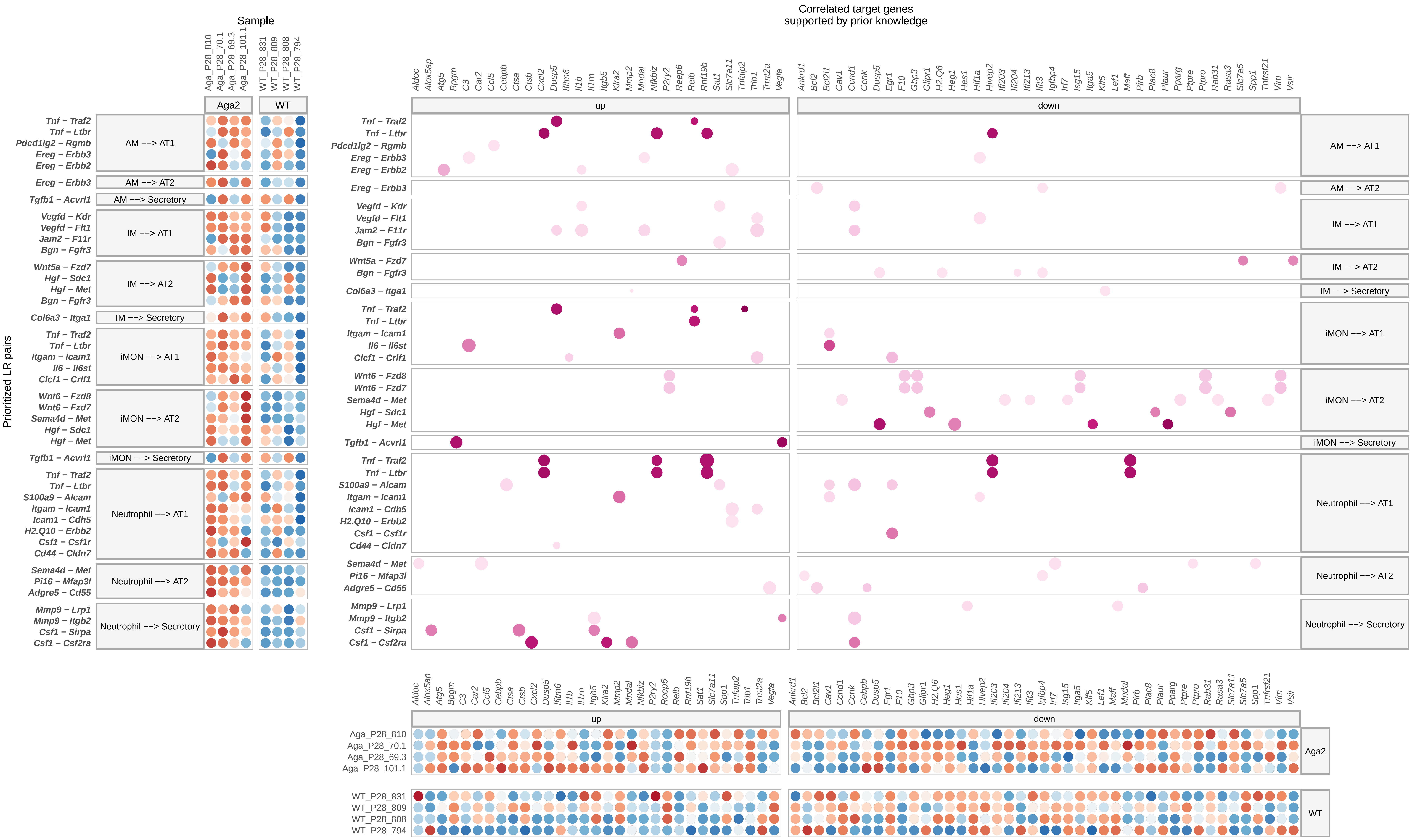
Sample
Correlated target genes supported by prior knowledge
Prioritized LR pairs
Aga_P28_810
Aga_P28_70.1
Aga_P28_69.3
Aga_P28_101.1
WT_P28_831
WT_P28_809
WT_P28_808
WT_P28_794
Aga2
WT
up
down
AM --> AT1
AM --> AT2
AM --> Secretory
IM --> AT1
IM --> AT2
IM --> Secretory
iMON --> AT1
iMON --> AT2
iMON --> Secretory
Neutrophil --> AT1
Neutrophil --> AT2
Neutrophil --> Secretory
Tnf – Traf2
Tnf – Ltbr
Pdcd1lg2 – Rgmb
Ereg – Erbb3
Ereg – Erbb2
Tgfb1 – Acvrl1
Vegfd – Kdr
Vegfd – Flt1
Jam2 – F11r
Bgn – Fgfr3
Wnt5a – Fzd7
Hgf – Sdc1
Hgf – Met
Col6a3 – Itga1
Itgam – Icam1
Il6 – Il6st
Clcf1 – Crlf1
Wnt6 – Fzd8
Wnt6 – Fzd7
Sema4d – Met
S100a9 – Alcam
Icam1 – Cdh5
H2.Q10 – Erbb2
Csf1 – Csf1r
Cd44 – Cldn7
Pi16 – Mfap3l
Adgre5 – Cd55
Mmp9 – Lrp1
Mmp9 – Itgb2
Csf1 – Sirpa
Csf1 – Csf2ra
Aldoc
Alox5ap
Atg5
Bpgm
C3
Car2
Ccl5
Cebpb
Ctsa
Ctsb
Cxcl2
Dusp5
Ifitm6
Il1b
Il1rn
Itgb5
Klra2
Mmp2
Mndal
Nfkbiz
P2ry2
Reep6
Relb
Rnf19b
Sat1
Slc7a11
Spp1
Tnfaip2
Trib1
Trmt2a
Vegfa
Ankrd1
Bcl2
Bcl2l1
Cav1
Ccnd1
Ccnk
Egr1
F10
Gbp3
Glipr1
H2.Q6
Heg1
Hes1
Hif1a
Hivep2
Ifi203
Ifi204
Ifi213
Ifit3
Igfbp4
Irf7
Isg15
Itga5
Klf5
Lef1
Maff
Pirb
Plac8
Plaur
Pparg
Ptpre
Ptpro
Rab31
Rasa3
Slc5a5
Slc7a5
Spp1
Tnfrsf21
Vim
Vsir

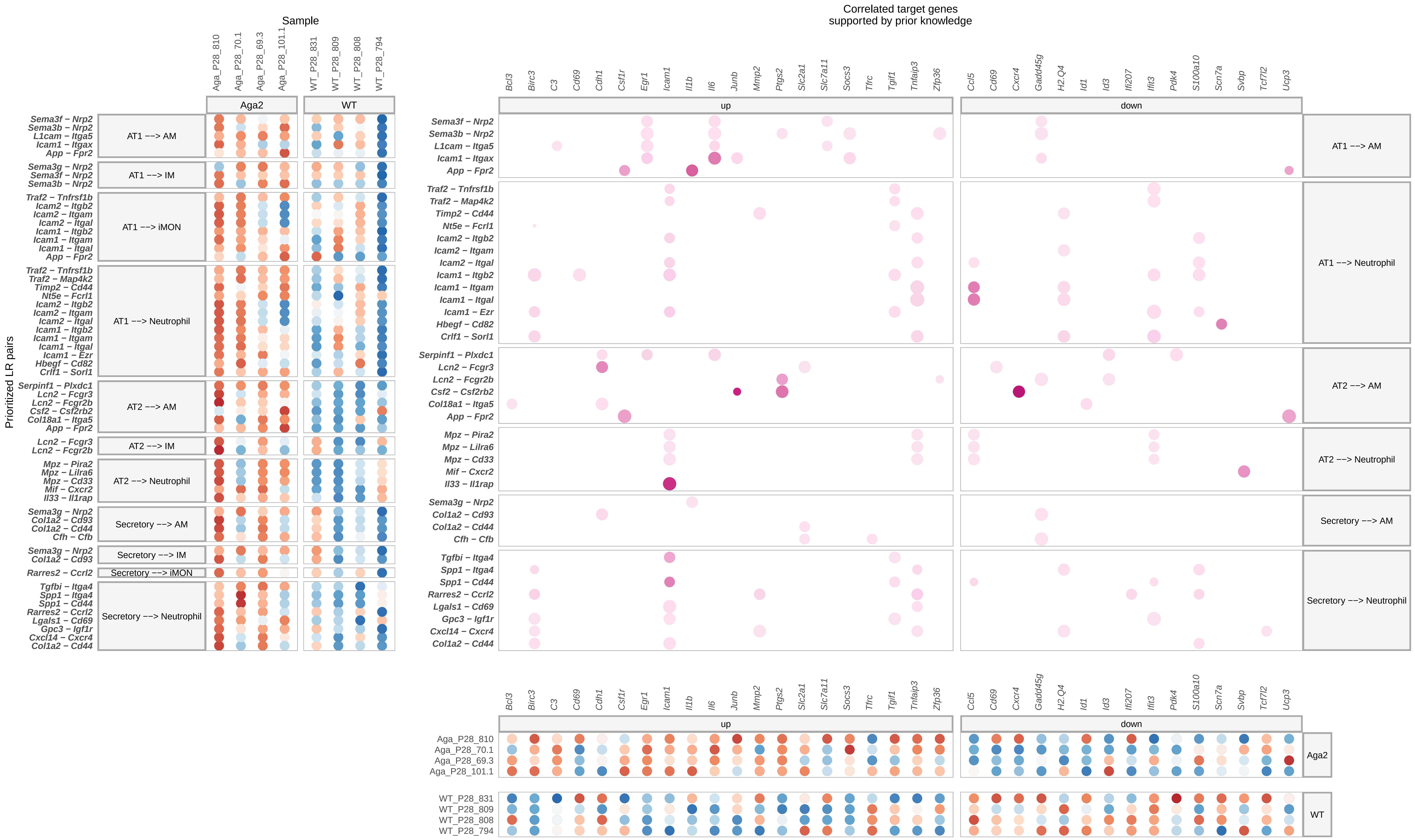
Sample
Aga_P28_810
Aga_P28_70.1
Aga_P28_69.3
Aga_P28_101.1
WT_P28_831
WT_P28_809
WT_P28_808
WT_P28_794
Aga2
WT
Prioritized LR pairs
Sema3f − Nrp2
Sema3b − Nrp2
L1cam − Itga5
Icam1 − Itgax
App − Fpr2
AT1 −−> AM
Sema3g − Nrp2
Sema3f − Nrp2
Sema3b − Nrp2
AT1 −−> IM
Traf2 − Tnfrsf1b
Icam2 − Itgb2
Icam2 − Itgam
Icam2 − Itgal
Icam1 − Itgb2
Icam1 − Itgam
Icam1 − Itgal
App − Fpr2
AT1 −−> iMON
Traf2 − Tnfrsf1b
Traf2 − Map4k2
Timp2 − Cd44
Nt5e − Fcrl1
Icam2 − Itgb2
Icam2 − Itgam
Icam2 − Itgal
Icam1 − Itgb2
Icam1 − Itgam
Icam1 − Itgal
Icam1 − Ezr
Hbegf − Cd82
Crlf1 − Sorl1
AT1 −−> Neutrophil
Serpinf1 − Plxdc1
Lcn2 − Fcgr3
Lcn2 − Fcgr2b
Csf2 − Csf2rb2
Col18a1 − Itga5
App − Fpr2
AT2 −−> AM
Lcn2 − Fcgr3
Lcn2 − Fcgr2b
AT2 −−> IM
Mpz − Pira2
Mpz − Lilra6
Mpz − Cd33
Mif − Cxcr2
Il33 − Il1rap
AT2 −−> Neutrophil
Sema3g − Nrp2
Col1a2 − Cd93
Col1a2 − Cd44
Cfh − Cfb
Secretory −−> AM
Sema3g − Nrp2
Col1a2 − Cd93
Secretory −−> IM
Rarres2 − Ccrl2
Secretory −−> iMON
Tgfbi − Itga4
Spp1 − Itga4
Spp1 − Cd44
Rarres2 − Ccrl2
Lgals1 − Cd69
Gpc3 − Igf1r
Cxcl14 − Cxcr4
Col1a2 − Cd44
Secretory −−> Neutrophil
Correlated target genes
supported by prior knowledge
Bcl3
Birc3
C3
Cd69
Cdh1
Csf1r
Egr1
Icam1
Il1b
Il6
Junb
Mmp2
Ptgs2
Slc2a1
Slc7a11
Socs3
Tfrc
Tgif1
Tnfaip3
Zfp36
Ccl5
Cd69
Cxcr4
Gadd45g
H2.Q4
Id1
Id3
Ifi207
Ifit3
Pdk4
S100a10
Scn7a
Svbp
Tcf7l2
Ucp3
up
down
Sema3f − Nrp2
Sema3b − Nrp2
L1cam − Itga5
Icam1 − Itgax
App − Fpr2
AT1 −−> AM
Traf2 − Tnfrsf1b
Traf2 − Map4k2
Timp2 − Cd44
Nt5e − Fcrl1
Icam2 − Itgb2
Icam2 − Itgam
Icam2 − Itgal
Icam1 − Itgb2
Icam1 − Itgam
Icam1 − Itgal
Icam1 − Ezr
Hbegf − Cd82
Crlf1 − Sorl1
AT1 −−> Neutrophil
Serpinf1 − Plxdc1
Lcn2 − Fcgr3
Lcn2 − Fcgr2b
Csf2 − Csf2rb2
Col18a1 − Itga5
App − Fpr2
AT2 −−> AM
Mpz − Pira2
Mpz − Lilra6
Mpz − Cd33
Mif − Cxcr2
Il33 − Il1rap
AT2 −−> Neutrophil
Sema3g − Nrp2
Col1a2 − Cd93
Col1a2 − Cd44
Cfh − Cfb
Secretory −−> AM
Tgfbi − Itga4
Spp1 − Itga4
Spp1 − Cd44
Rarres2 − Ccrl2
Lgals1 − Cd69
Gpc3 − Igf1r
Cxcl14 − Cxcr4
Col1a2 − Cd44
Secretory −−> Neutrophil
Bcl3
Birc3
C3
Cd69
Cdh1
Csf1r
Egr1
Icam1
Il1b
Il6
Junb
Mmp2
Ptgs2
Slc2a1
Slc7a11
Socs3
Tfrc
Tgif1
Tnfaip3
Zfp36
Ccl5
Cd69
Cxcr4
Gadd45g
H2.Q4
Id1
Id3
Ifi207
Ifit3
Pdk4
S100a10
Scn7a
Svbp
Tcf7l2
Ucp3
up
down
Aga_P28_810
Aga_P28_70.1
Aga_P28_69.3
Aga_P28_101.1
Aga2
WT_P28_831
WT_P28_809
WT_P28_808
WT_P28_794
WT

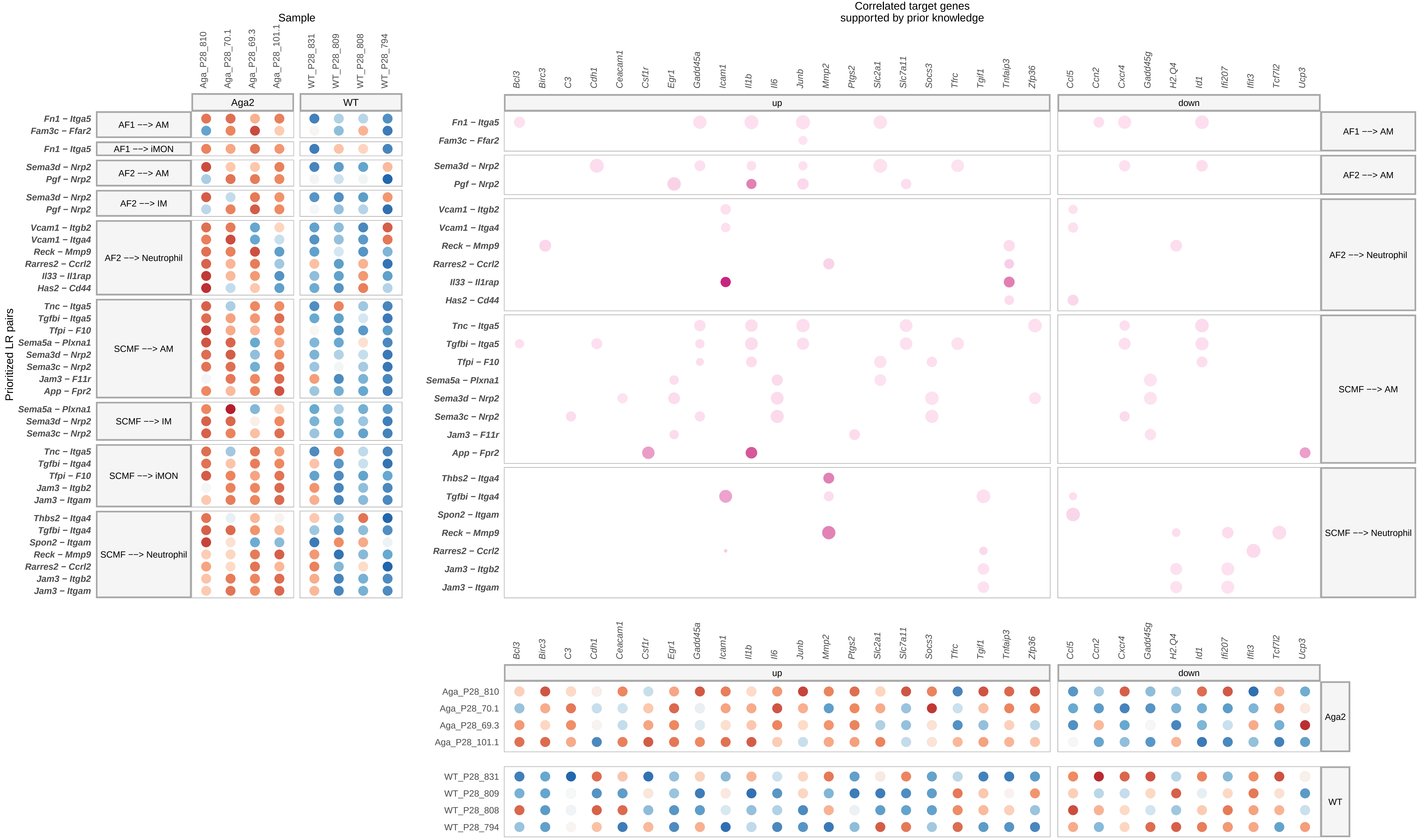

Sample
Aga2
WT
Aga_P28_810
Aga_P28_70.1
Aga_P28_69.3
Aga_P28_101.1
WT_P28_831
WT_P28_809
WT_P28_808
WT_P28_794
Prioritized LR pairs
AF1 --> AM
AF1 --> iMON
AF2 --> AM
AF2 --> IM
AF2 --> Neutrophil
SCMF --> AM
SCMF --> IM
SCMF --> iMON
SCMF --> Neutrophil
Fn1 − Itga5
Fam3c − Ffar2
Sema3d − Nrp2
Pgf − Nrp2
Vcam1 − Itgb2
Vcam1 − Itga4
Reck − Mmp9
Rarres2 − Ccrl2
Il33 − Il1rap
Has2 − Cd44
Tnc − Itga5
Tgfbi − Itga5
Tfpi − F10
Sema5a − Plxna1
Sema3c − Nrp2
Jam3 − F11r
App − Fpr2
Tgfbi − Itga4
Jam3 − Itgb2
Jam3 − Itgam
Thbs2 − Itga4
Spon2 − Itgam
Correlated target genes
supported by prior knowledge
up
down
Bcl3
Birc3
C3
Cdh1
Ceacam1
Csf1r
Egr1
Gadd45a
Icam1
Il1b
Il6
Junb
Mmp2
Ptgs2
Slc2a1
Slc7a11
Socs3
Tfrc
Tgif1
Tnfaip3
Zfp36
Ccl5
Ccn2
Cxcr4
Gadd45g
H2.Q4
Id1
Ifi207
Ifit3
Tcf7l2
Ucp3

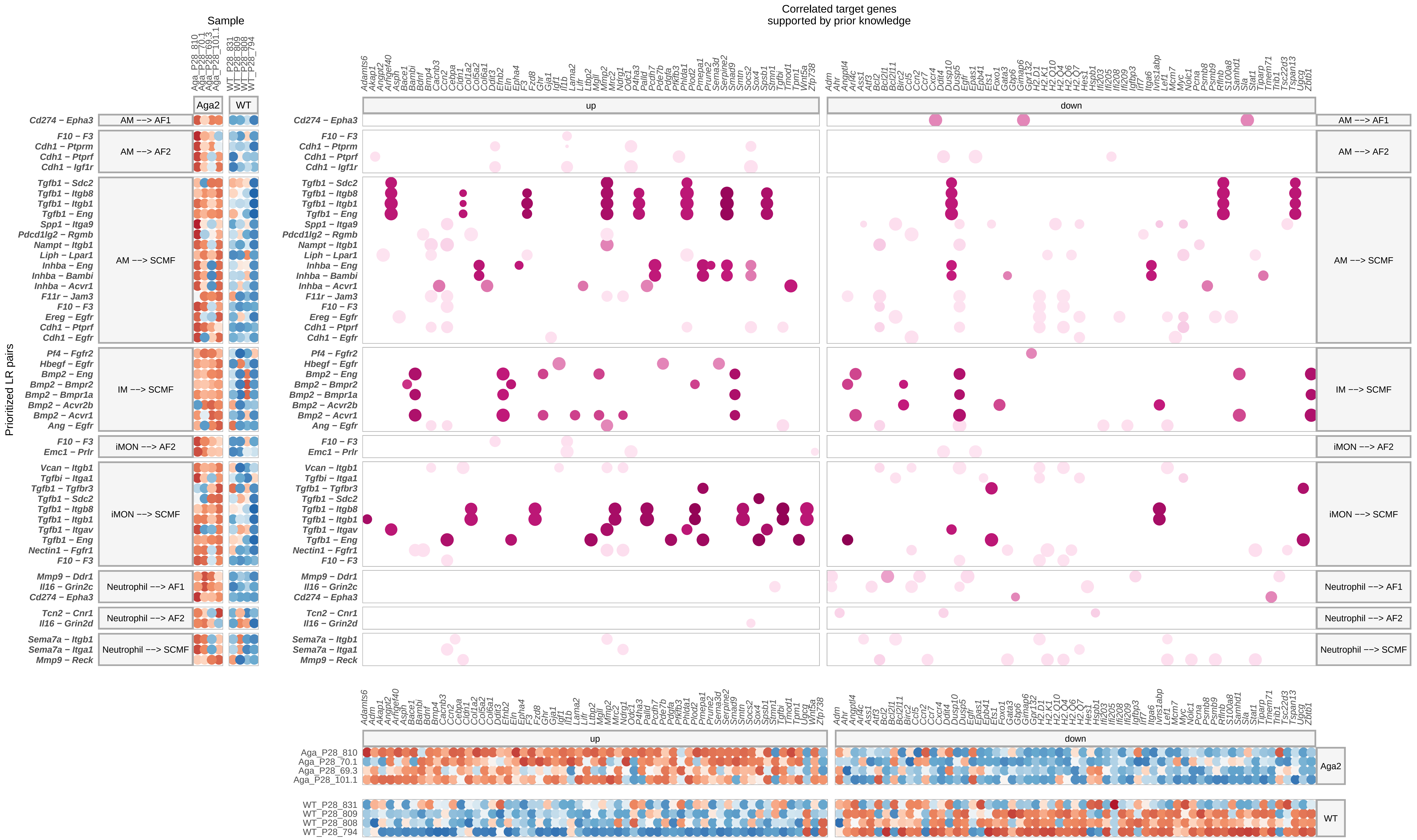

Sample
Correlated target genes
supported by prior knowledge
Aga_P28_810
Aga_P28_70.1
Aga_P28_69.3
Aga_P28_101.1
WT_P28_831
WT_P28_809
WT_P28_808
WT_P28_794
Aga2
WT
up
down
Prioritized LR pairs
AM --> AF1
AM --> AF2
AM --> SCMF
IM --> SCMF
iMON --> AF2
iMON --> SCMF
Neutrophil --> AF1
Neutrophil --> AF2
Neutrophil --> SCMF
Cd274 − Epha3
F10 − F3
Cdh1 − Ptprm
Cdh1 − Ptprf
Cdh1 − Igf1r
Tgfb1 − Sdc2
Tgfb1 − Itgb8
Tgfb1 − Itgb1
Tgfb1 − Eng
Spp1 − Itga9
Pdcd1lg2 − Rgmb
Nampt − Itgb1
Liph − Lpar1
Inhba − Eng
Inhba − Bambi
Inhba − Acvr1
F11r − Jam3
F10 − F3
Ereg − Egfr
Cdh1 − Ptprf
Cdh1 − Egfr
Pf4 − Fgfr2
Hbegf − Egfr
Bmp2 − Eng
Bmp2 − Bmpr2
Bmp2 − Bmpr1a
Bmp2 − Acvr2b
Bmp2 − Acvr1
Ang − Egfr
F10 − F3
Emc1 − Prlr
Vcan − Itgb1
Tgfbi − Itga1
Tgfb1 − Tgfbr3
Tgfb1 − Sdc2
Tgfb1 − Itgb8
Tgfb1 − Itgb1
Tgfb1 − Itgav
Tgfb1 − Eng
Nectin1 − Fgfr1
F10 − F3
Mmp9 − Ddr1
Il16 − Grin2c
Cd274 − Epha3
Tcn2 − Cnr1
Il16 − Grin2d
Sema7a − Itgb1
Sema7a − Itga1
Mmp9 − Reck
Adamts6 Akap1 Angpt2 Arhgef40 Asph Bace1 Bambi Bdnf Bmp4 Cacnb3 Ccn2 Cebpa Cldn1 Col1a2 Col5a2 Col6a1 Ddit3 Efnb2 Eln Epha4 F3 Fzd8 Ghr Gja1 Igf1 Il1b Lama2 Lifr Ltbp2 Mgll Mmp2 Mrc2 Ndrg1 Odc1 P4ha3 Palld Pcdh7 Pde7b Pdgfa Pfkfb3 Phlda1 Plod2 Pmepa1 Prune2 Sema3d Serpine2 Smad9 Smtn Socs2 Sox4 Spsb1 Stmn1 Tgfbi Tmod1 Tpm1 Wnt5a Zfp738
Adm Ahr Angptl4 Arl4c Ass1 Atf3 Bcl2l1 Bcl2l11 Birc2 Ccl5 Ccn2 Ccr7 Cxcr4 Ddit4 Dusp10 Dusp5 Egfr Epas1 Epb41 Ets1 Foxo1 Gata3 Gbp6 Gimap6 Gpr132 H2.D1 H2.K1 H2.Q10 H2.Q4 H2.Q6 H2.Q7 Hes1 Hspb1 Ifi203 Ifi205 Ifi208 Ifi209 Igfbp3 Irf7 Itga6 Ivns1abp Lef1 Mcm7 Myc Nolc1 Pcna Psmb8 Psmb9 Rflnb S100a8 Samhd1 Sla Stat1 Tiparp Tmem71 Trib1 Tsc22d3 Tspan13 Ugcg Zbtb1
